# Polynitroxylated PEGylated hemoglobin protects pig brain neocortical gray and white matter after traumatic brain injury and hemorrhagic shock

**DOI:** 10.3389/fmedt.2023.1074643

**Published:** 2023-02-21

**Authors:** Jun Wang, Yanrong Shi, Suyi Cao, Xiuyun Liu, Lee J. Martin, Jan Simoni, Bohdan J. Soltys, Carleton J. C. Hsia, Raymond C. Koehler

**Affiliations:** ^1^ Department of Anesthesiology and Critical Care Medicine, Johns Hopkins University, Baltimore, MD, United States; ^2^ Department of Pathology, Johns Hopkins University, Baltimore, MD, United States; ^3^ AntiRadical Therapeutics LLC, Sioux Falls, SD, United States

**Keywords:** controlled cortical impact, frontal lobe damage, hemoglobin-based oxygen carrier, dendrite/axon rescue, neuroprotection

## Abstract

Polynitroxylated PEGylated hemoglobin (PNPH, aka SanFlow) possesses superoxide dismutase/catalase mimetic activities that may directly protect the brain from oxidative stress. Stabilization of PNPH with bound carbon monoxide prevents methemoglobin formation during storage and permits it to serve as an anti-inflammatory carbon monoxide donor. We determined whether small volume transfusion of hyperoncotic PNPH is neuroprotective in a porcine model of traumatic brain injury (TBI) with and without accompanying hemorrhagic shock (HS). TBI was produced by controlled cortical impact over the frontal lobe of anesthetized juvenile pigs. Hemorrhagic shock was induced starting 5 min after TBI by 30 ml/kg blood withdrawal. At 120 min after TBI, pigs were resuscitated with 60 ml/kg lactated Ringer's (LR) or 10 or 20 ml/kg PNPH. Mean arterial pressure recovered to approximately 100 mmHg in all groups. A significant amount of PNPH was retained in the plasma over the first day of recovery. At 4 days of recovery in the LR-resuscitated group, the volume of frontal lobe subcortical white matter ipsilateral to the injury was 26.2 ± 7.6% smaller than homotypic contralateral volume, whereas this white matter loss was only 8.6 ± 12.0% with 20-ml/kg PNPH resuscitation. Amyloid precursor protein punctate accumulation, a marker of axonopathy, increased in ipsilateral subcortical white matter by 132 ± 71% after LR resuscitation, whereas the changes after 10 ml/kg (36 ± 41%) and 20 ml/kg (26 ± 15%) PNPH resuscitation were not significantly different from controls. The number of cortical neuron long dendrites enriched in microtubules (length >50 microns) decreased in neocortex by 41 ± 24% after LR resuscitation but was not significantly changed after PNPH resuscitation. The perilesion microglia density increased by 45 ± 24% after LR resuscitation but was unchanged after 20 ml/kg PNPH resuscitation (4 ± 18%). Furthermore, the number with an activated morphology was attenuated by 30 ± 10%. In TBI pigs without HS followed 2 h later by infusion of 10 ml/kg LR or PNPH, PNPH remained neuroprotective. These results in a gyrencephalic brain show that resuscitation from TBI + HS with PNPH protects neocortical gray matter, including dendritic microstructure, and white matter axons and myelin. This neuroprotective effect persists with TBI alone, indicating brain-targeting benefits independent of blood pressure restoration.

## Introduction

Traumatic brain injury (TBI) produces an immediate mechanical injury to brain tissue followed by the secondary injury that evolves over acute, subacute, and chronic stages instigated by imbalances in neurotransmitter signal processing and connectivity, mitochondrial dysfunction, oxidative stress, and neuroinflammation (1–5). With moderate or severe TBI, early treatment focuses on maintaining mean arterial blood pressure (MABP) and minimizing brain swelling to mitigate intracranial hypertension. Both factors influence the cerebral pressure gradient, which can be particularly critical in TBI victims because autoregulation-maintained cerebral blood flow can be impaired in some patients (6). Several studies have demonstrated that clinical outcome correlates with maintenance of cerebral perfusion pressure in the range where the limited post-traumatic vascular autoregulatory mechanisms remain operative (7–9). Furthermore, some TBI victims suffer systemic organ-wide multi-trauma accompanied by hemorrhage shock (HS) that can exacerbate brain injury by reducing MABP and blood O_2_-carrying capacity due to the loss of red blood cells. Consequently, an ideal therapeutic for TBI + HS should have pleiotropic multi-target properties that serve not only as a resuscitation fluid to restore perfusion pressure and O_2_ carrying capacity, but also to directly protect the brain from secondary injury cascades associated with oxidative stress and neuroinflammation.

Polynitroxylated PEGylated hemoglobin (PNPH, SanFlow™) is in advanced preclinical development both as a nanobiotherapeutic and as a third-generation blood substitute (10, 11). It is a manufactured ∼8 nm nanoparticle with bovine hemoglobin as the protein center and with bound nitroxide groups and conjugated PEG. Here, we propose that PNPH meets the criteria for a pleiotrophic multitarget resuscitation fluid for several reasons. First, PNPH has the ability to carry O_2_ into the microcirculation and, owing to its high affinity for O_2_, will unload O_2_ preferentially in tissue with low PO_2_ driven by diffusion gradient. Moreover, because PNPH is in the plasma compartment, it can deliver O_2_ to capillaries that are poorly perfused by red blood cells and improve the homogeneous delivery of O_2_. Second, the PEGylation increases hemoglobin's oncotic pressure and allows it to be used as a small volume resuscitation fluid, which is of logistical benefit for use in the field and military settings (12, 13). The high oncotic pressure also serves to limit cerebral edema after TBI (12, 13). Third, the PEGylation provides the additional benefit of increasing the molecular radius so that it is not filtered in the renal glomeruli or in other vascular beds (14), thereby extending its circulating half-life and reducing the need for frequent infusions. The restrained extravasation also reduces abluminal scavenging of nitric oxide by hemoglobin and associated vasoconstriction (15–17), which was an adverse effect of earlier generations of cell-free crosslinked hemoglobin solutions (18) that failed in clinical trials (19). Fourth, PNPH is stored with carbon monoxide (CO) bound to the heme to prevent autooxidation to methemoglobin (metHb) and thus extend its shelf life. Furthermore, when infused into the circulation, the CO is released and re-equilibrates with red blood cell-based hemoglobin and other heme moieties in the tissue. The resulting low partial pressure of CO is known to exert anti-inflammatory effects as shown by the use of other CO donors (20, 21). Fifth, PNPH has nitroxide moieties conjugated to lysine residues on the hemoglobin molecule and these moieties react with superoxide anion (22, 23), effectively serving as a plasma-based superoxide dismutase to protect the endothelium from oxidative stress and leukocyte adhesion (24, 25). Interestingly, PNPH also has been shown to protect cultured neurons from the toxic effects of native hemoglobin and elevated glutamate (26). Thus, any extravasation of PNPH at a site of blood-brain barrier breakdown after TBI will be less toxic than native hemoglobin and could even exert direct neuroprotective effects against excitotoxicity by virtue of its antioxidant properties. One concern with the use of cell-free hemoglobin is that it undergoes oxidation to the ferric state, wherein it can be further oxidized to the highly reactive ferryl state in the presence of H_2_O_2_ (27). However, the nitroxide moieties also possess peroxidase activity that limits oxidation to the ferryl state (28, 29), thereby limiting toxicity of hemoglobin outside of the red blood cell. Hence, PNPH has many characteristics suitable both for fluid resuscitation from TBI + HS and protecting the brain vasculature and parenchyma.

Previously, PNPH was found to help maintain dilation of pial arteries, improve collateral blood flow, and reduce infarct volume in ischemic stroke (30). In a mouse model of TBI + HS, PNPH was more effective than crystalloid or whole blood in restoring arterial pressure and that resuscitation with PNPH ameliorated cerebral edema and neurodegeneration in hippocampus (12, 13, 26). Likewise, in a guinea pig model of TBI + HS, resuscitation with PNPH was superior to lactated Ringer's (LR) solution in restoring arterial pressure, preserving hippocampal viable neurons and reducing loss of white matter (31). Based on these encouraging results in rodents, testing the efficacy of PNPH in a large animal model was now deemed warranted for possible clinical translation.

Pigs are used increasingly as a large animal for TBI studies (32). In most studies, they typically range in age from 4 days (neonatal model) (33), to 1 month (toddler model) (34), and to 35–45 kg (pre-pubertal juvenile model) (35); the latter are considered pediatric models. Because sexually mature adult domestic pigs are more difficult to accommodate logistically in most laboratories, minipig strains provide an alternative adult model (36, 37), but their financial cost is considerably greater than domestic pigs. Here, we used 3-month-old juvenile pigs (approximately 30 kg) in our model of TBI + HS for testing the therapeutic efficacy of PNPH at doses of 10 or 20 ml/kg (300–600 ml for a 30-kg pig). Like humans, pigs have a large gyrencephalic brain and abundant white matter. Others have established a swine model of TBI + HS induced by controlled cortical impact (CCI) combined with 2 h of HS followed by therapeutic resuscitation for evaluating hematologic and cerebral biochemical effects at 6-h survival (38–41) and neurologic deficits with longer-term survival (35, 42). We adapted this model for neuropathological evaluation at 4 days after injury to interrogate the effects of PNPH on evolving brain damage in survivors.

Our first objective was to evaluate two doses of PNPH during the resuscitation from TBI + HS on histopathological outcomes, including cortical contusion injury volume, loss of subcortical white matter, impairment of axonal transport as a functional marker of axonal injury, dendritic damage as assessed by microtubule-associated protein disintegration, and microglia morphology as a marker of neuroinflammation. The experimental protocol for TBI + HS utilized CCI followed by 2 h of arterial hypotension induced by 30 ml/kg hemorrhage. Pigs were then resuscitated with 60 ml/kg infusion of LR or 10 or 20 ml/kg of PNPH. This study was intended to inform whether a single infusion of PNPH at resuscitation from TBI + HS could sustain improvement of histological markers of secondary brain injury over a 4-day period. The 4-day recovery period allows time for secondary neuronal injury processes and inflammation to develop. Furthermore, having a neuroprotective agent that mitigates acute secondary injury and sustains brain viability for several days is also of clinical importance in some military arenas and in remote civilian settings, wherein blood typing and storage are not always available and patient transport to advanced critical care units may take several days.

Our second objective was to determine whether PNPH has neuroprotective efficacy after TBI in the absence of induced HS. In previous work in mouse and guinea pig (26, 31), PNPH infusion restored mean arterial pressure (MAP) more quickly than infusion of LR. Thus, the neuroprotection seen in these earlier studies may have been attributed to the hemodynamic benefits of PNPH. To determine whether protection can occur independent of effects on MAP, we conducted a second experiment to test the efficacy of PNPH infusion after TBI alone.

## Methods

### Surgical preparation

All procedures on pigs were approved by the Johns Hopkins University Animal Care and Use Committee and by the Animal Care and Use Review Office of the US Army Medical Research and Materiel Command for Award Number W81XWH-19-C-0022 (Fort Detrick, MD). In conducting research using animals, the investigators adhered to the Animal Welfare Act Regulations and other Federal statutes relating to animals and experiments involving animals and the principles set forth in the current version of the Guide for the Care and Use of Laboratory Animals, National Research Council.

**Figure 1:**
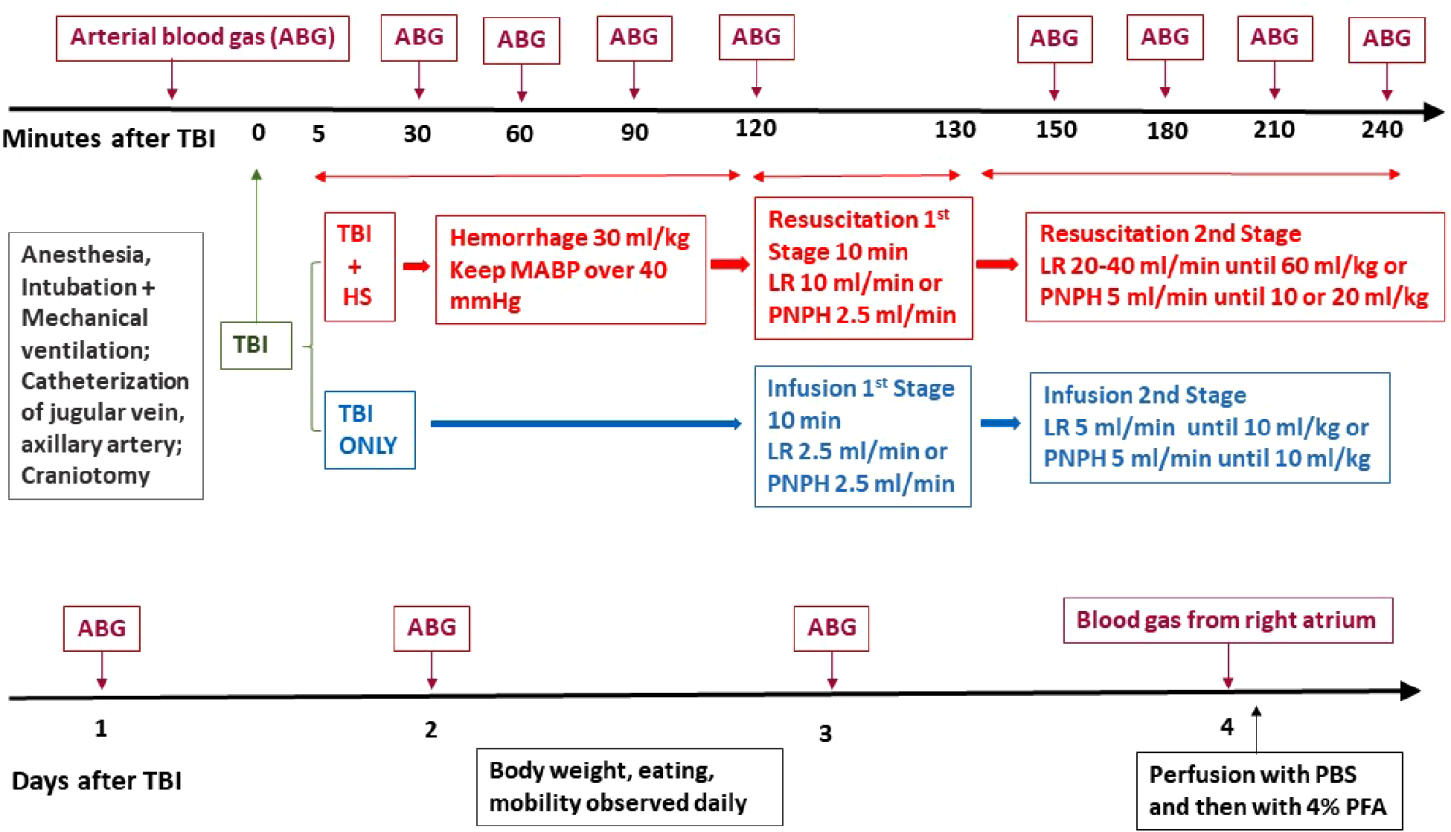
Schematic outline of time line of protocol for TBI + hemorrhagic shock (HS) and TBI only experiments on pigs during the first 4 h of recovery (top) and during the 4-day survival period (bottom). ABG, arterial blood gas; CCI, controlled cortical impact; LR, lactated Ringer's solution; PBS, phosphate-buffered saline; PFA, paraformaldehyde.

Because there can be sex differences in the response to TBI (43, 44) and TBI in the young and in military personnel is more prevalent in males (45), the study was conducted in male pigs. A total of 48 pigs weighing 28 ± 2 kg and approximately 3 months of age were used in the overall study. The experimental protocols for the TBI + HS experiment and the TBI alone experiment are delineated in Figure 1. The pigs were sedated with intramuscular injection of Telazol (50 mg/ml tiletamine and 50 mg/ml zolazepam, 4.4 mg/kg each component), ketamine 2.2 mg/kg and xylazine 2.2 mg/kg. Isoflurane (4% in 30% O_2_) was administered *via* face mask to produce an anesthetic depth for oral intubation of the trachea. After a surgical plane of anesthesia was achieved, as assessed by the lack of limb withdrawal to hoof pinching and by looseness of muscle tone in the jaw, anesthesia was maintained with 2% isoflurane in approximately 30% O_2_ with mechanical ventilation of the lungs. The antibiotic Baytril 10 mg/kg (100 mg/ml) was injected intramuscularly. Surgery was conducted using aseptic techniques. Through a 5-cm neck incision, an external jugular vein was isolated by blunt dissection. The vein was ligated and a catheter was advanced toward the heart and secured with another ligature. For arterial catheterization, we chose the axillary artery because occlusion of the carotid artery could limit cerebral blood flow after TBI and catheterization of the femoral artery can limit use of the hindlimb. An incision was made in the axilla, and the axillary artery was isolated, ligated, and cannulated with a flexible polyvinyl catheter that minimized kinking. The arterial and venous catheters were tunneled subcutaneously to the back of the neck, where they exited through a small incision. Pigs were able to bear weight on the forelimb and ambulate on the day after surgery.

### Traumatic brain injury

Pigs were injected with fentanyl 20 µg/kg bolus and followed by continuous infusion of 20 µg/kg/h through the jugular vein catheter while maintained on isoflurane anesthesia. They were placed in a stereotactic head holder that was placed on the base of the impactor frame to prevent movement of the head during impact. The CCI model was similar to that described by others (38). A 20 mm diameter craniotomy was made over the right frontal cortex centered 1 cm anterior to the bregma and 1 cm lateral to the midline. The dura remained intact. Once the craniotomy was complete, the isoflurane concentration was decreased to 1%. At 5 min before the TBI, the isoflurane was stopped while fentanyl continued to be infused. TBI was produced with a CCI device (Custom Design and Fabrication, Inc., Sandston, VA, USA) that used a linear electric motor accelerator to control an impactor with a 15 mm diameter and a rounded tip. We used a 10 cm inward displacement of the dura at a velocity of 5 m/s for a duration of 50 ms. After CCI, gel foam was inserted into the craniotomy and sealed with tissue glue. The scalp incision was closed, and lidocaine hydrochloride cream (2.0%) was topically applied to all surgical incisions. The pigs remained sedated by infusing 0.1 mg/kg midazolam while continuing the infusion of 20 µg/kg/h fentanyl. For postoperative analgesia, they received a subcutaneous injection of sustained-release buprenorphine (0.12 mg/kg). They typically became responsive and were extubated at 3–5 h after TBI and were placed in a recovery cage for additional physiology monitoring for the first 4 h after TBI. When they were able to stand, they were returned to their pen in the central housing facility and continued to be observed for full mobility, which usually occurred within 6 h after TBI. Pigs were observed at least twice per day over the 4-day recovery period and were assessed for appetite, urination, defecation, mobility and level of awareness.

### Hemorrhagic hypotension

In the first experiment with TBI + HS, hemorrhage was produced by withdrawing 30 ml/kg of blood starting 5 min after TBI. Because very low mean arterial pressure (MAP) will decrease coronary blood flow and cardiac contractility, hemorrhage sometimes needs to be briefly interrupted to prevent loss of cardiac function (42). In our protocol, blood withdrawal was temporarily stopped when MAP fell to 35–40 mmHg and resumed when MAP began to recover above 40 mmHg. When the targeted amount of blood had been removed, blood withdrawal ceased and MAP was allowed to spontaneously increase above 40 mmHg. Thus, we used a variable rate of blood withdrawal to achieve a fixed volume of hemorrhage while attempting to avoid MAP < 35 mmHg and minimize the risk of cardiogenic shock.

### Treatment groups

In the first experiment, pigs were randomized to groups undergoing TBI followed by 2 h of hemorrhagic hypotension and then fluid resuscitation with 60 ml/kg lactated Ringer's (LR) solution (*n* = 7), 10 ml/kg PNPH (*n* = 8), or 20 ml/kg PNPH (*n* = 8). In the second experiment, pigs undergoing TBI without hemorrhagic hypotension were randomized to fluid treatment with 10 ml/kg LR solution (*n* = 6) or 10 ml/kg PNPH (*n* = 9) starting 2 h after TBI. A sham-operated group underwent catheterization and craniotomy, but without CCI or hemorrhage (*n* = 5). Because the craniotomy itself can potentially cause inflammation in the meninges and in cortex, brains from a naïve group without surgery were also collected (*n* = 5).

Fluid treatment commenced 2 h after TBI in those with and without induced hemorrhagic hypotension. Because PEGylation conveys hyper-oncotic properties to the molecule, smaller infusion volumes of PNPH are needed to restore MAP than LR. In addition, rapid fluid resuscitation can cause a rapid increase in preload and afterload, which can lead to pulmonary edema when cardiac contractility is already compromised by low perfusion pressure. Because cardiac output can be reduced and cerebral edema worsened with rapid LR infusion, others recommend using stepwise increases in infusion rates (39). Therefore, we performed fluid resuscitation with stepwise increasing infusion rates. In the group resuscitated with LR after hemorrhage, infusion started at a rate of 10 ml/min for 5 min (50 ml), then 20 ml/min for 22.5 min (additional 450 ml), and lastly at 60 ml/min until 60 ml/kg was infused (twice the hemorrhage volume). In the group without hemorrhage and treated with LR, infusion was stopped when a total of 10 ml/kg was infused. For the PNPH infusion groups, resuscitation commenced with a priming dose of LR infused at a rate of 10 ml/min for 5 min followed by PNPH infusion at a rate of 2.5 ml/min for 10 min and then at 5 ml/min until the targeted dose of 10 or 20 ml/kg was achieved. In the pigs that underwent hemorrhage, an additional LR infusion was performed at the end of the infusion period until the total of PNPH + LR equaled 30 ml/kg (the hemorrhage volume). If mean arterial pressure dropped below 40 mmHg during HS or early resuscitation, epinephrine was infused to maintain coronary perfusion pressure and avoid irreversible shock.

Throughout the first 4 h after TBI or sham surgery, arterial blood pressure was recorded and arterial blood samples (0.5 ml) were obtained hourly. The samples were analyzed for pH, blood gases, Hb concentration, metHb, COHb, and glucose with an ABL800Flex blood gas machine (Radiometer Inc., Brea, CA USA). In the group receiving PNPH, the remaining blood sample was centrifuged to obtain plasma, which was then analyzed for Hb concentration. Rectal temperature also was monitored.

On day 1, 2, 3 and 4 of recovery, a 1 ml blood sample was withdrawn from the arterial catheter for analysis of total blood Hb concentration, metHb, and COHb. The plasma Hb was also analyzed in the PNPH groups. If the arterial catheter was not patent, then samples were drawn from the jugular vein. Values from arterial and venous blood were combined for statistical analysis because the Hb concentration, metHb, and COHb from the 2 sites are expected to be similar. On day 4, piglets were deeply anesthetized and perfused transcardially with cold phosphate-buffered saline (PBS) followed by cold 4% paraformaldehyde. The thoracic aorta was clamped to direct fixative to the upper body. The quality of ongoing and final tissue fixation was judged by neck and facial muscle firmness. The heads were removed and placed in 4% paraformaldehyde to allow for *in situ* post-fixation of the brain within the skull overnight. Then the brains were carefully dissected and placed in a solution of 4% paraformaldehyde and 30% sucrose in PBS at 4°C. Using the optic chiasm and pons as landmarks, the brain was cut into 3 blocks. The anterior third block was cut into 50 µm sections with a sliding microtome and serially allocated into three 12-well plates and repeating the order 1–36.

### Lesion volume and white matter volume analysis

Evaluation of neuropathology was performed with blinding to treatment group. Histologic sections from the anterior third of the brain, encompassing the frontal lobes, were used to estimate the ipsilateral and contralateral hemisphere volume of structurally normal appearing cerebral cortex and the volume of peri-lesion subcortical white matter. Every 12th serial coronal section was stained with 0.1% Luxol fast blue followed by 0.1% cresyl violet staining after dehydration in a series of increasing alcohol concentrations. Luxol fast blue staining was used to identify white matter. Cresyl violet was used to counterstain tissue sections for lesion identification and neocortex loss. Using Image J software, we traced the area of each hemisphere excluding the contusion injury (cavity plus pale cresyl violet stained tissue) and the area of white matter in each hemisphere. We calculated the volume by summing section area × 600 µm spacing between sections along the entire axial length of the anterior third of the cerebral hemisphere. The percentage of contusion injury volume was calculated 100 (left–right)/left hemisphere volume. The percentage of white matter volume loss was calculated as 100 (left–right)/left white matter volume. For this analysis, 15–20 brain sections were used to calculate the percentage of contusion injury volume and the percentage of white matter volume loss for each brain.

### Immunohistochemistry and immunofluorescence

Immunostaining for amyloid precursor protein (APP) was used as a surrogate marker for impairment of axonal transport because it is known to accumulate when transport function in axons is blocked (46). Neuronal somatodendritic integrity was assessed by immunostaining for the cytoskeletal protein microtubule-associated protein-2 (MAP-2). Assessment of microglia morphology was performed with immunostaining for Iba-1. Immunohistochemistry was performed on 50 µm free-floating sections under moderate shaking. Before staining, the sections were incubated 30 min in 0.3% hydrogen peroxide to quench endogenous peroxidases. After three washing steps in 0.1 M phosphate buffer (pH 7.4), non-specific antibody binding sites were blocked with using 10% normal goat serum. Different free-floating sections were incubated overnight at 4 °C with anti-MAP-2 (1 : 500, monoclonal mouse-IgG; Millipore, MAB3418), anti-APP (1 : 500, monoclonal mouse-IgG; Millipore, MAB348), or anti-Iba-1 (1 : 500, polyclonal rabbit-IgG; Wako, 019-19741) in 5% normal goat serum. After several washes, sections were incubated for 2 h at room temperature with secondary antibodies. For APP and MAP-2 staining, biotinylated anti-mouse-IgG, 1 : 500, Vector, BA9200 was used, and the streptavidin/horseradish peroxidase detection was performed according to the manufacturer's recommendations. These sections were incubated with the substrate diaminobenzidine (DAB, D3939; Sigma-Aldrich Company, St. Louis, MO, United States) for 10 min at room temperature. For Iba-1 staining, Alexa Fluor 594 sary antibody (1:500, Invitrogen, #A-11037) was used. Immunofluorescent images were obtained with a Leica microscope (model DMi8 with THUNDER Imager 3D software).

### Stereological measurement for APP-positive particles

Stereological measurements were made by using a Nikon Eclipse 90i microscope (Nikon, Tokyo, Japan) attached to a Qimage Retiga-2000R camera, which was connected to a workstation with Stereo Investigator software (Version 10; MicroBrightField, Williston, VT, USA). Every 12th section was examined, placing the analyzed sections 600 μm apart. Using a 2× objective, we traced the entire area of subcortical white matter for outlining the region of interest to be used in the stereological analysis. Then we counted the APP-positive particles under a 40× objective. We identified particles as being a brown dot that first came into focus within the optical dissector counting frame. The counting frame was 150 × 150 μm with a grid size of 3,000 × 3,000 μm. The dissector height was set at 10 μm with an upper and lower guard zone of 1 μm. This analysis used 11–14 coronal sections and 98–176 sampling fields to ensure that at least 100 particles were counted per brain (range 118–1086) to obtain the estimate of the total number of APP-positive particles within the entire subcortical white matter area.

### MAP-2 immunohistochemistry analysis

Processes stained for MAP-2 had a more fragmented appearance after TBI, indicative of disruption of microtubules. As a measure of dendrite integrity, we counted the number of MAP-2–immunopositive processes with a length >50 µm within the plane of sectioning of the 50 µm-thick sections. This 50 µm threshold length was based on a preliminary survey of the images in the TBI brains where many of the fragmented dendrites were <50 µm in length. For this analysis, adjacent images of the entire cortical mantle of the gyrus lateral to the contused gyrus were obtained at 40× and the image tiles were stitched together to obtain a single composite image. For each brain, 10–12 sections spaced 600 µm apart were analyzed and the number of long processes per coronal section were averaged. Counting was performed on approximately 100 fields of view (0.25 mm^2^ area each) per section spread over a 5 × 5 mm area of the cortical mantle. For each brain, approximately 1,000 fields were used to obtain the number of MAP-2 immunostained processes >50 µm per section.

### Iba-1 immunohistochemistry analysis

Iba-1 immunofluorescent images were obtained on 3 coronal sections for each brain. On each section, 3 fields of view at 40× power were analyzed in the peri-lesion area. For each brain, an average value was obtained from the 9 fields of view. The total number of Iba-1–positive cells were counted to quantify the number of microglia/macrophages in the peri-lesion area. In addition, we quantified three different kinds of morphologies of these cells. Those with a small cell body area and long processes were regarded as ramified microglia. Those with a larger cell body area and shorter processes were regarded as hypertrophic microglia. Those which almost had no long, thin processes were regarded as fully activated bushy microglia. Because the hypertrophied and bushy morphologies were not always distinguishable, these two morphologies were combined for statistical purposes.

### Statistical analysis

All data were analyzed by GraphPad Prism version 6 statistical software. Measurement data were expressed by mean ± standard deviation. Statistical analysis included all pigs that completed the protocol and survived for brain perfusion and fixation: The number of pigs in each group was: 5 naïve, 5 sham surgery, 5 TBI + HS + 60 ml/kg LR, 4 TBI + HS + 10 ml/kg, PNPH, 5 TBI + HS + 20 ml/kg PNPH, 6 TBI alone + 10 ml/kg LR, and 6 TBI alone + 10 ml/kg PNPH. For histologic analysis, the naïve and sham groups were combined into a single control group of 10 pigs since all of the histologic values were similar in each group. The TBI + HS experiment and the TBI alone experiment were analyzed with separate one-way analysis of variance (ANOVA) for each experiment. If the F value was significant, then we used the Holm-Sidak procedure to keep the family-wise error at <0.05 for multiple comparisons. In the TBI + HS experiment, there were 5 comparisons of interest. We compared the combined Naïve + Sham group with (1) TBI + HS + LR, (2) TBI + HS + 10 ml/kg PNPH and (3) TBI + HS + 20 ml/kg PNPH; and we compared TBI + HS + LR with (4) TBI + HS + 10 ml/kg PNPH and (5) TBI + HS + 20 ml/kg PNPH. In the TBI alone experiment, there were 3 comparisons of interest. We compared the combined naïve + Sham group with (1) the TBI + LR group and (2) the TBI + 10 ml/kg PNPH group; and we compared the TBI + LR group with (3) the TBI + 10 ml/kg PNPH group. For physiologic measurements, some missing values occurred because of difficulty in drawing arterial blood samples or because of technical problems with the blood gas analyzer. In addition, some sham-operated pigs regained consciousness more quickly than the TBI pigs and were too active to obtain MAP and blood sample measurements at the 120–240 min time points, resulting in *n* = 2–3 at these times. Because of the small sample size, the sham group was not included in the statistical comparisons for MAP and blood sample analysis.

## Results

### Enrollment and attrition

In the TBI + HS experiment, 33 pigs were enrolled in the TBI + HS study and assigned to an intention-to-treat group prior to the day of surgery. Of these, 24 survived and were used for neuropathologic analysis. These included: (1) 5 naïve pigs perfused with fixative to provide normal brain histology, (2) 5 survivors from sham surgery, (3) 5 survivors from TBI + HS + LR resuscitation (mortality = 2 of 7 pigs: one pig died during surgery from presumed arrhythmia, 1 pig died 4 h after TBI), (4) 4 survivors from 10 ml/kg PNPH resuscitation (mortality = 4 of 8 pigs: one pig died during hemorrhage before the start of resuscitation; two pigs were hypotensive during resuscitation and died at 2.5–4 h after TBI; one pig completed the resuscitation protocol, but its arterial catheter accidently got pulled out when the pig was waking up from anesthesia and moving its legs, which resulted in a second episode of hypotension; this pig was humanely euthanized), (5) 5 survivors from 20 ml/kg PNPH resuscitation (mortality = 3 of 8 pigs: one pig died during hemorrhage before the start of resuscitation; two pigs were hypotensive during resuscitation and died at 2.5–3 h after TBI). Thus, among the groups destined to undergo TBI + HS, 14 of 23 pigs completed the protocol.

In the TBI alone experiment, 15 pigs were enrolled and 12 survived and were used for neuropathologic analysis. These included: (1) 6 survivors from TBI + LR infusion (no mortality) and (2) 6 survivors from TBI + 10 ml/kg PNPH infusion (mortality = 3 of 9: two pigs had severe hypotension leading to cardiac arrest during infusion; one pig failed to fully regain consciousness).

### Physiological results with TBI + HS

**Figure 2:**
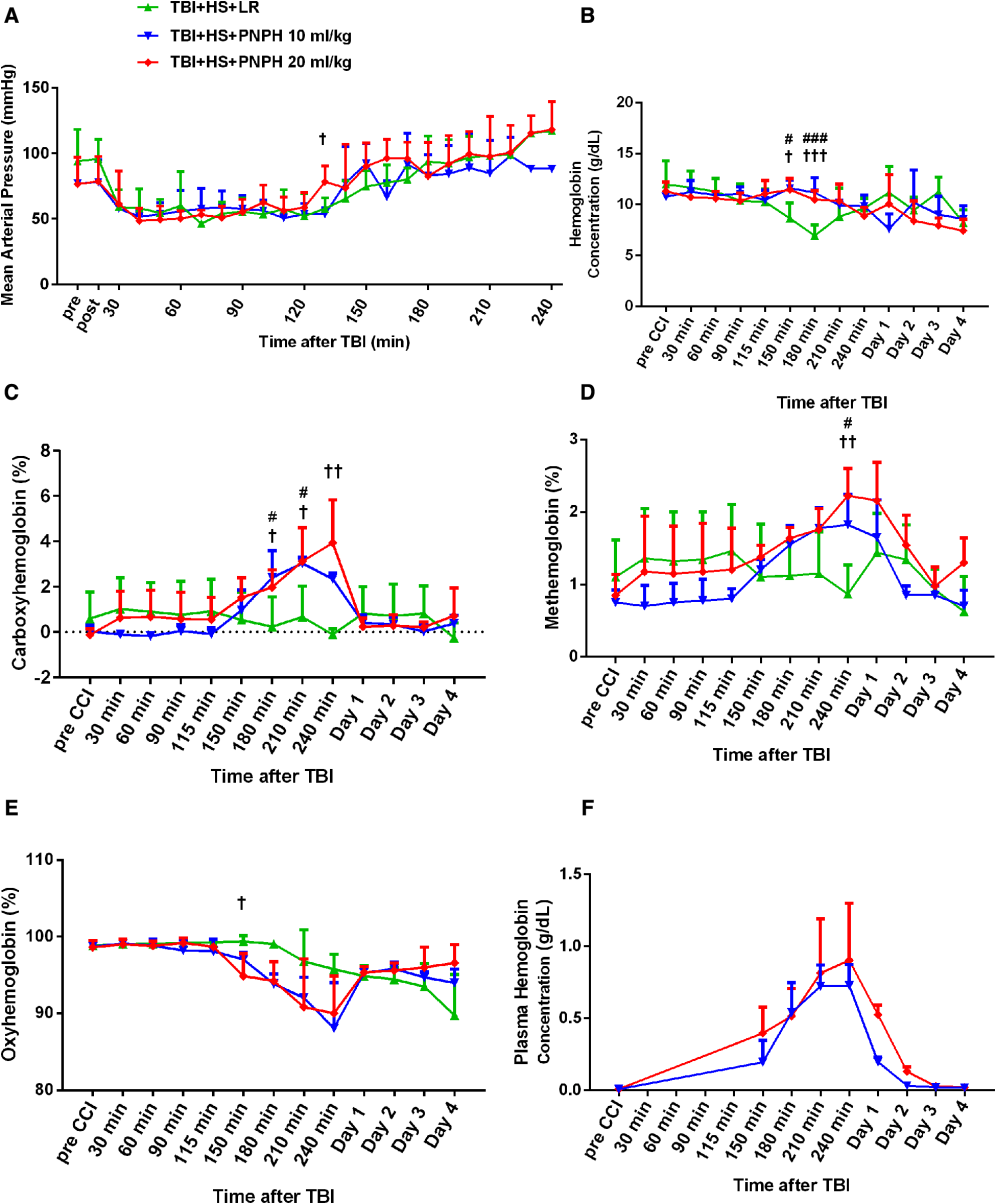
Time course of mean arterial pressure (**A**), hemoglobin concentration (**B**), percent of carboxyhemoglobin (**C**), percent of methemoglobin (**D**), and percent of oxygenated hemoglobin (**E**) in whole blood for groups of pigs subjected to TBI and 120 min of hemorrhagic shock (HS) followed by infusion of 60 ml/kg lactated Ringer's (LR) solution, 10 ml/kg PNPH, or 20 ml/kg PNPH. Sample sizes per time point were 3–5 for LR group, 3–4 for 10 ml/kg PNPH group, and 4–5 for 20 ml/kg PNPH group (smaller sample sizes occurred at 180–240 min when arterial blood samples could no longer be drawn because the pig was too active or the subcutaneously-routed catheter was inoperable). Individual data are shown in Supplemental Figures 1–5. PNPH infusion produced increases in plasma hemoglobin concentration that subsided over 1–2 days (**F**). Values are means ± SD. ^#^*P* < 0.05, ^###^*P* < 0.001 TBI + HS + LR vs. TBI + HS + 10 ml/kg PNPH; ^†^*P* < 0.05, ^††^*P* < 0.01 TBI + HS + LR vs. TBI + HS + 20 ml/kg PNPH. Significance determined by one-way ANOVA and the Holm-Sidak procedure for multiple comparisons.

The time course of physiologic data is presented for the survivors from each group in Figure 2 and the individual data are shown at each time point in Supplementary Figures S1–S5. All TBI + HS groups subjected to HS had a similar level of MAP at the time of TBI and had a similar level of hypotension during the 2 h of HS (Figure 2A). In all TBI + HS groups, MAP recovered to their pre-injury levels within 30–60 min of the start of fluid resuscitation. All groups had further increases in MAP to approximately 100 mmHg as pigs awakened at variable times. Thus, the different resuscitation fluid composition and volumes were well matched to yield a similar recovery of MAP. This allowed comparisons of potential neuroprotective effects to be independent of the recovery of MAP.

The concentration of hemoglobin (Hb) decreased during the fluid resuscitation with LR at 150 and 180 min after TBI (30 and 60 min of fluid infusion) compared to the PNPH groups, which were infused at a slower rate (Figure 2B). PNPH is nearly saturated with carbon monoxide (CO) to prevent metHb formation during storage. During the infusion of PNPH, CO is released from PNPH and equilibrates with native Hb. The total blood COHb (plasma plus red blood cell Hb) increased progressively during PNPH infusion but remained below 4% (Figure 2C). COHb recovered to normal levels by Day 1. PNPH infusion also resulted in a significant increase in metHb by 240 min after TBI compared to the LR group (Figure 2D), but the elevation was only about 1% metHb and resolved by 1–2 days. Together, the increase in COHb and metHb will act to decrease the fraction of total Hb that was carrying oxygen (FHbO2). Although there was a trend for FHbO2 to decrease at 210 and 240 min when COHb and metHb were at their peak levels, the decrease in FHbO2 was variable and did not significantly differ from values in the group resuscitated with LR at these time points (Figure 2E). The concentration of Hb in the plasma increased to 0.71 ± 0.16 g/dl after infusion of 10 ml/kg PNPH and then declined to 0.21 ± 0.03 g/dl on Day 1 and to 0.03 ± 0.02 g/dl on Day 2 (Figure 2F). After 20 ml/kg PNPH infusion, plasma Hb concentration increased to 0.84 ± 0.40 g/dl and declined to 0.52 ± 0.07 g/dl on Day 1 and to 0.13 ± 0.04 g/dl on Day 2 as PNPH was slowly cleared from the circulation. Thus, a significant amount of PNPH was retained in the circulation over the first two days of recovery.

### Neuropathologic results with TBI + HS

**Figure 3:**
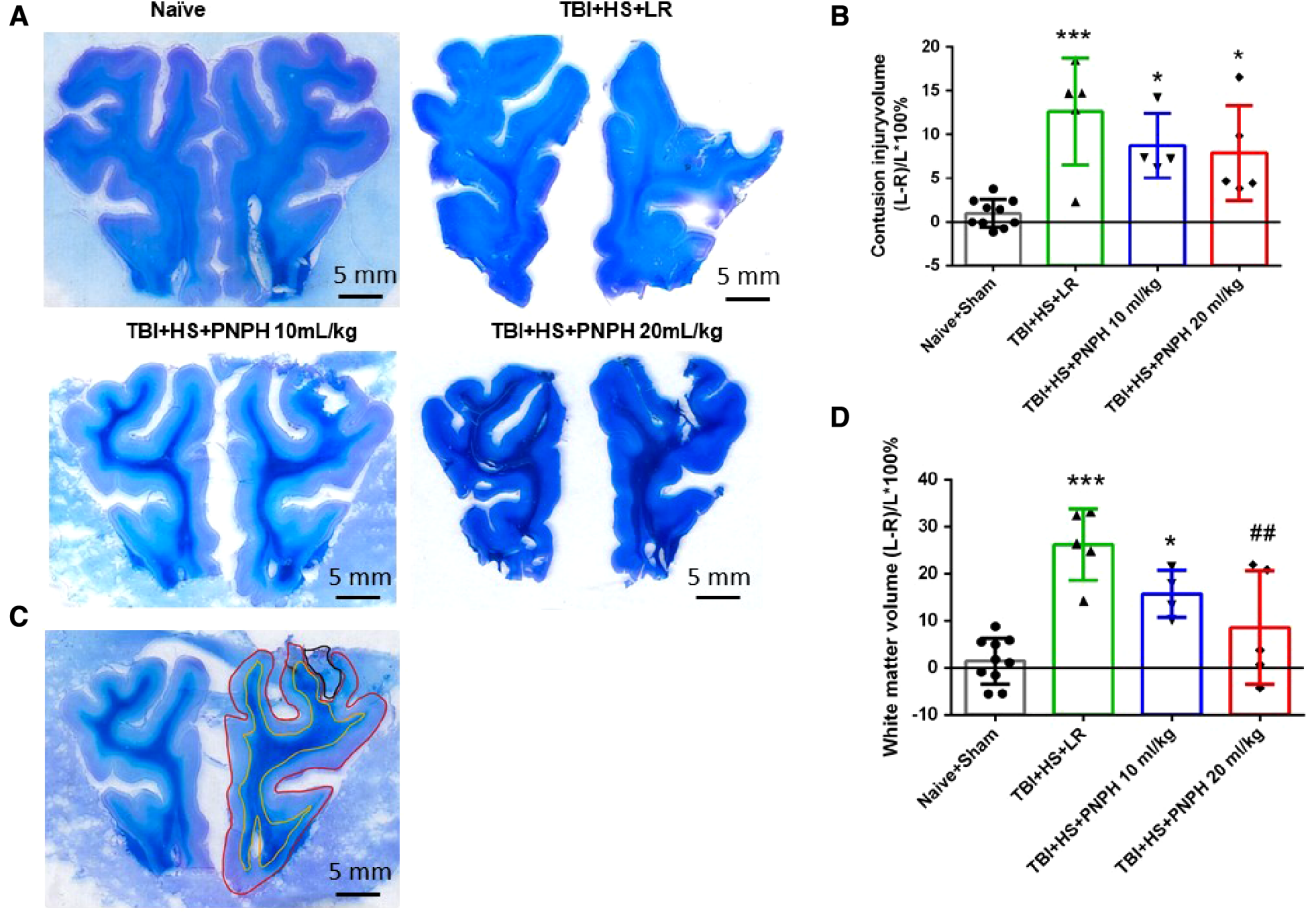
Effect of PNPH on contusion injury volume and volumetric white matter loss in frontal lobe after TBI + hemorrhagic shock (HS). (**A**) Representative coronal sections (both hemispheres, top is superior) of frontal lobe of normal pig brain and brains 4 days after TBI and resuscitation with 60 ml/kg lactated Ringer's (LR) solution and 10 and 20 ml/kg PNPH. Gray matter is visualized with cresyl violet staining and subcortical white matter myelin is visualized with Luxol fast blue staining. (**B**) Area delineated by red trace is the entire histologically normal hemisphere, area delineated by the yellow trace is the subcortical white matter, and area circled by the black trace is the contusion site. (**C**) Contusion injury volume (intact tissue difference of left minus right hemisphere as a percentage of left hemisphere volume contralateral to the injury site) of pigs subjected to TBI + HS followed by resuscitation at 120 min with LR (*n *= 5) or 10 ml/kg PNPH (*n *= 4) and 20 ml/kg PNPH (*n *= 5). The normal variation in the difference of left minus right hemisphere volume as a percentage of left hemisphere volume is shown for the combined Naïve (*n* = 5) and Sham (*n* = 5) group. (**D**) Volume of white matter loss in the same brains was calculated as the difference between left minus right subcortical white matter volume normalized by the left subcortical white matter volume (contralateral volume). The loss of white matter seen in the LR group was attenuated in the 20 ml/kg PNPH group. Mean ± SD and individual data for each pig are shown. *P < 0.05, ***P < 0.001 vs. combined Naïve and Sham groups. ^##^*P* < 0.05 vs. LR group determined by one-way ANOVA and the Holm-Sidak procedure for multiple comparisons.

Representative coronal brain sections stained with Luxol fast blue followed by cresyl violet are shown from a naïve pig and TBI + HS pigs resuscitated with LR and PNPH (Figure 3A). At 4 days of recovery, cavitation with some remnants of hemorrhage were evident in the cerebral cortex and underlying white matter. The areas used for measuring intact hemisphere volume and white matter volume are illustrated by the red and yellow traces, respectively, in Figure 3B. Contusion injury area measurements included the cavity and tissue with atypical sparse cresyl violet staining. The remaining area of histologically normal tissue in the injured hemisphere was subtracted from the area of the contralateral hemisphere and summed across the frontal lobe to estimate the contusion lesion volume, which was normalized by the contralateral hemisphere volume. Because the Sham group had minor differences between the volume of each hemisphere volume, pigs in this group were combined with the Naïve group as a single control group for statistical analysis. One-way ANOVA indicated a significant overall effect of treatment among the four groups [*F* (3,20) = 10.463; *P* < 0.001]. Contusion injury volume averaged 13%, 9%, and 8% of the homotypic contralateral hemisphere volume in the TBI + HS groups resuscitated with 60 ml/kg LR, 10 ml/kg PNPH, and 20 ml/kg PNPH, respectively (Figure 3C). Contusion injury volume exhibited considerable variability and no significant differences were noted among the 3 TBI + HS groups with TBI + HS with the Holm-Sidak procedure.

White matter loss, relative to the homotypic contralateral white matter volume, averaged 26% in the frontal lobe in the TBI group resuscitated with LR, 16% in the group resuscitated with 10 ml/kg PNPH, and 9% in the group resuscitated with 20 ml/kg PNPH (Figure 3D). The overall effect of group intervention was significant by one-way ANOVA [*F* (3,20) = 13.233; *P* < 0.001]. Multiple comparisons with the Holm-Sidak procedure indicated that the level in the 20 ml/kg PNPH group was significantly less than the level in the LR group (*P* = 0.006) but was not significantly different than the measurements in the Naïve + Sham group (*P* = 0.18). This result suggests that PNPH protects white matter in a gyrencephalic brain where there is an abundance of subcortical white matter.

**Figure 4:**
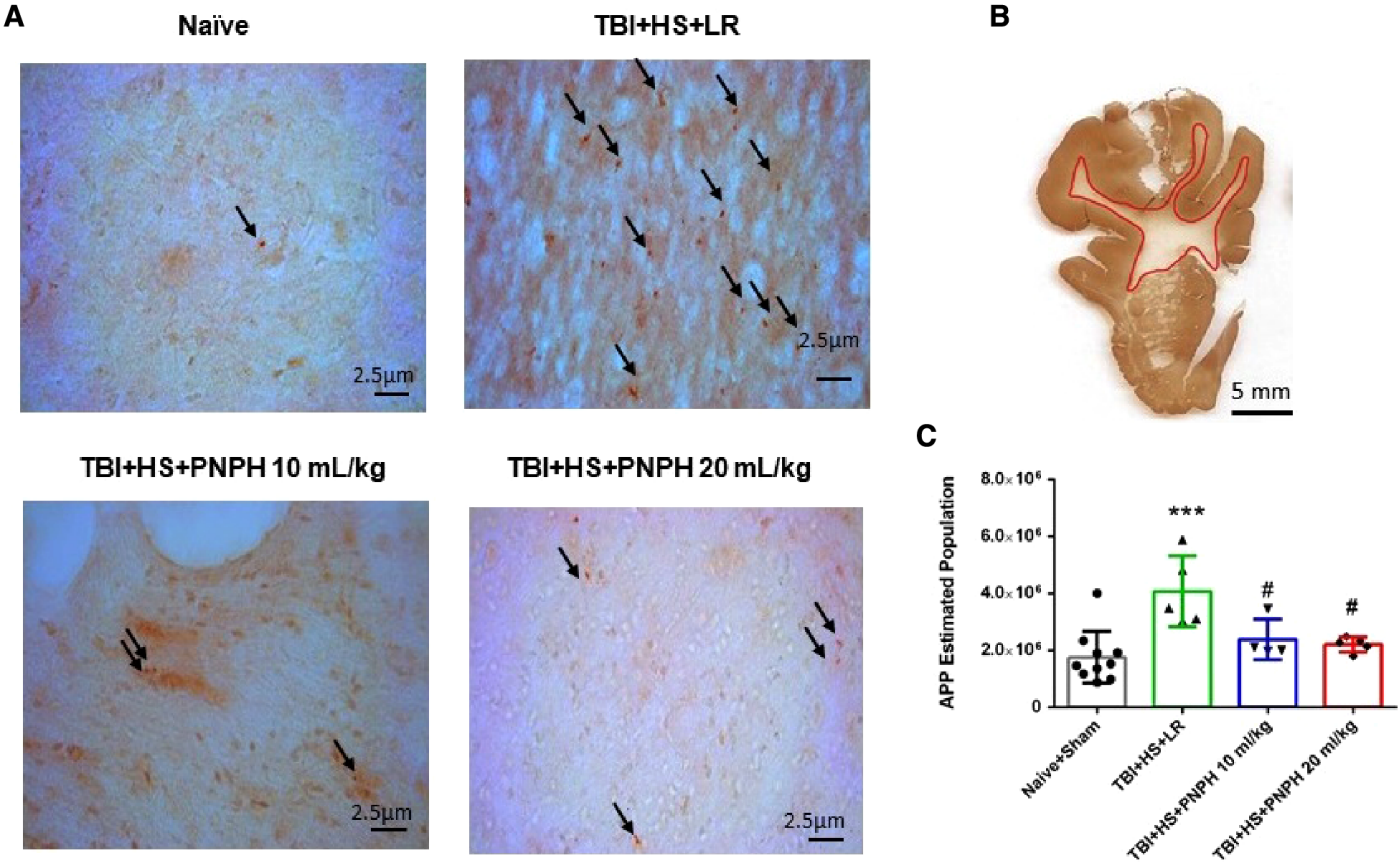
Effect of PNPH on amyloid precursor protein (APP) punctate accumulation after TBI + hemorrhagic shock (HS). (**A**) Representative images of APP immunostaining from naïve pig and TBI + HS pigs resuscitated with 60 ml/kg lactated Ringer's (LR) solution or 10 ml/kg PNPH and 20 ml/kg PNPH. Arrows point to particles of APP accumulation. (**B**) The area of subcortical white used in the analysis is illustrated with the red trace. (**C**) The number of APP particles throughout subcortical white matter was estimated with the optical fractionator method of unbiased stereology and are plotted for the combined Naïve + Sham group (*n* = 10) and for TBI + HS groups resuscitated with 60 ml/kg LR (*n* = 5), 10 ml/kg PNPH (*n* = 4), and 20 ml/kg PNPH (*n* = 5). Both doses of PNPH blunted the increase in APP particles seen in the LR group. Mean ± SD and individual data for each pig are shown. ***P < 0.001 vs. combined Naïve + Sham group. ^#^*P *< 0.05 vs. LR group determined by one-way ANOVA and the Holm-Sidak procedure for multiple comparisons.

Axonopathy was assessed by APP staining as interpreted by accumulation of punctate immunoreactivity in the white matter seen as brown particulate inclusions on DAB immunohistochemistry (Figure 4A). Using 11–14 equally spaced coronal sections per brain, unbiased stereological analysis was performed over the entire area of subcortical white matter. An example of the area included in the analysis is shown by the outlined red trace in Figure 4B. One-way ANOVA indicated a significant overall effect of group intervention [*F* (3,20) = 7.898; *P* < 0.001]. The Holm-Sidak procedure for multiple comparisons indicated a consistent increase in the estimated APP particles, from 1,749,836 ± 908,968 in the combined group Sham + Naïve group to 4,066,307 ± 1,244,399 in the TBI + HS group resuscitated with LR (*P *< 0.001, Figure 4C). In contrast, the estimated number of APP particles in groups resuscitated with 10 ml/kg PNPH (2,385,524 ± 715,346) and 20 ml/kg PNPH (2,201,756 ± 257,790) were not significantly different from the Sham + Naïve group (*P* > 0.5). Moreover, APP particle accumulation in the 10 ml/kg and 20 ml/kg PNPH groups were significantly less than that in the LR group (*P* < 0.05). These results indicate a significant rescue of neuronal axonal transport with PNPH resuscitation from TBI + HS.

**Figure 5:**
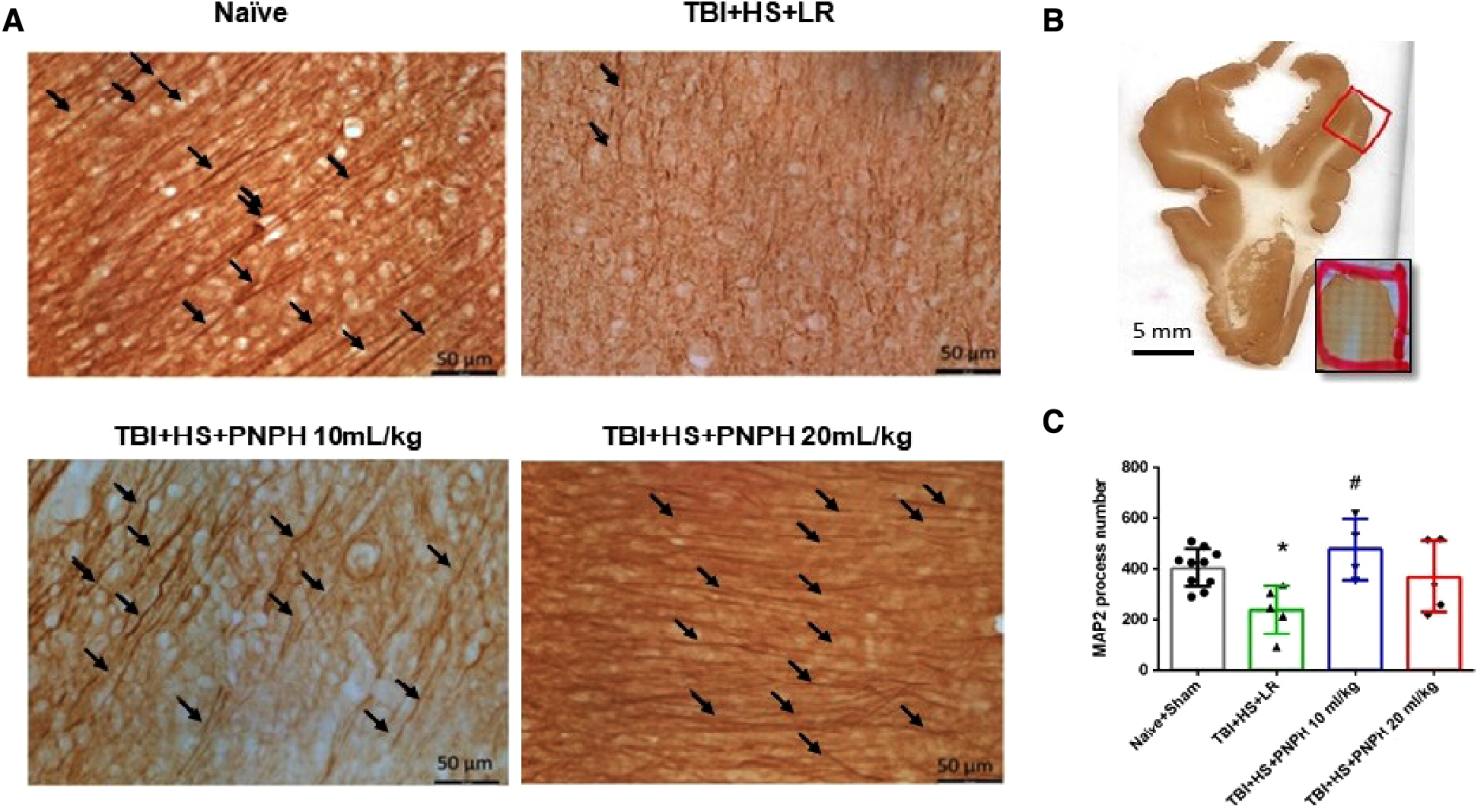
Effect of PNPH on MAP-2–identified gray matter neuronal dendrites after TBI + hemorrhagic shock (HS). (**A**) Representative images of MAP-2 immunostaining from naïve pig and TBI pigs resuscitated with 60 ml/kg lactated Ringer's (LR) solution or 10 ml/kg PNPH and 20 ml/kg PNPH. Arrows point to dendritic segments with >50 µm length from the gyrus of the cortex adjacent to the gyrus where the controlled cortical impact was produced. (**B**) The area used for MAP-2 analysis is outlined in the gyrus adjacent to the impacted gyrus. (**C**) The number of long dendrites in neocortex per coronal section averaged over 10–12 sections are plotted for the combined Naïve + Sham group (*n* = 10) and for TBI + HS groups resuscitated with 60 ml/kg LR (*n* = 5), 10 ml/kg PNPH (*n* = 4), and 20 ml/kg PNPH (*n* = 5). The dose of 10 ml/kg of PNPH blocked the decrease in long dendrites seen in the LR group. Mean ± SD and individual data for each pig are shown. *P < 0.05 vs. combined Naïve + Sham group, ^#^*P* < 0.05 vs. TBI + HS + LR group determined by one-way ANOVA and the Holm-Sidak procedure for multiple comparisons.

To assess neuronal dendrite integrity in neocortical gray matter of frontal lobe, we examined the number of long MAP-2–positive processes in the plane of the section. In Figure 5A, arrows point to the MAP-2–positive dendritic segments with >50 µm length. Counting was performed on 10–12 equally spaced coronal sections throughout the neocortex of the gyrus adjacent to the contusion as outlined in Figure 5B. One-way ANOVA indicated a significant overall effect of group intervention [*F* (3,20) = 4.615; *P* = 0.013]. The number of long dendritic processes per coronal section was reduced from 403 ± 75 in the Naïve + Sham group to 237 ± 95 in the TBI + HS + LR group (*P* < 0.05), whereas the number of long dendrites in the groups resuscitated with 10 ml/kg PNPH (406 ± 121) and 20 ml/kg PNPH (368 ± 139) were not significantly different from the Sham + Naïve group (Figure 5C). Furthermore, the value in the 10 ml/kg PNPH group was significantly greater than that seen with LR resuscitation (*P* < 0.05).

**Figure 6:**
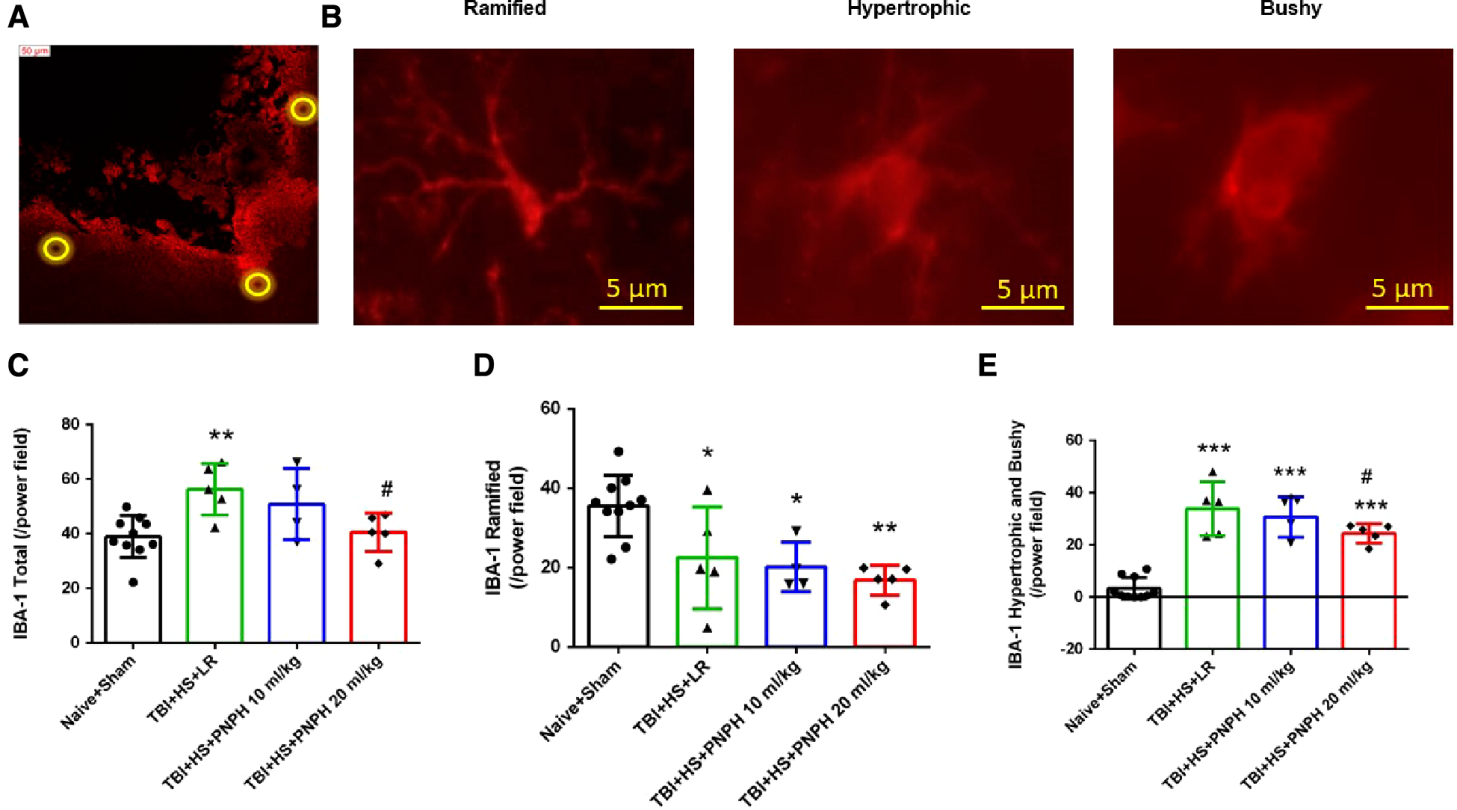
Effect of PNPH on microglial activation after TBI + hemorrhagic shock (HS). (**A**) Example of three fields selected for counting in the peri-lesion cortex on each side of the contusion and near the inner depth of the contusion. (**B**) Representative images of three morphologies of microglial cells: ramified, hypertrophic, and bushy. The number of Iba-1–positive cells (**C**), ramified Iba-1–positive cells (**D**), and hypertrophic plus bushy Iba-1–positive cells (**E**) are plotted for the combined Naïve + Sham group (*n* = 10) and for TBI + HS groups resuscitated with 60 ml/kg lactated Ringer's (LR) solution (*n* = 5), 10 ml/kg PNPH (*n* = 4), and 20 ml/kg PNPH (*n* = 5). The dose of 20 ml/kg PNPH attenuated the increased in microglia density and the number of hypertrophic/bushy microglia. Mean ± SD and individual data for each pig are shown. *P < 0.05, **P < 0.01, ***P < 0.001 vs. combined Naïve and Sham groups; ^#^*P *< 0.05 vs. LR group determined by one-way ANOVA and the Holm-Sidak procedure for multiple comparisons.

We next performed assessment of Iba1-positive cells to quantify microglial cell number and morphologic appearance in the cerebral cortex adjacent to the contusion. In each of 3 sections, we counted three fields of view as shown as Figure 6A, and obtained an average value from the total of 9 fields for each brain. We quantified the number of Iba-1–immunopositive cells, the number of immunopositive cells with a small soma and ramified process morphology, and the number of immunopositive cells with a large soma and shorter processes (hypertrophic microglia) or essentially no processes (bushy microglia). Examples are shown in Figure 6B. One-way ANOVA indicated a significant overall effect of group intervention for the total Iba-1–immunopositive cell number [*F* (3,20) = 5.187; *P* = 0.008], for the number of ramified microglia [*F* (3,20) = 7.360; *P* = 0.002], and for the number of hypertrophic and bushy microglia [*F* (3,20) = 35.002; *P* < 0.001]. For TBI + HS with LR resuscitation, the total Iba-1 immunopositive–cell number was significantly increased compared with Naïve + Sham group (*P* < 0.01). This increase was modestly but significantly reduced with 20 ml/kg PNPH resuscitation compared to LR resuscitation (*P* = 0.04) and not significantly different from the level in the Naïve + Sham group (Figure 6C). TBI + HS produced an overall decrease in the number of microglia with ramified processes without significant differences between the LR group and the two PNPH groups (Figure 6D). The decrease in ramified microglia was associated with an increase in the number of microglia with hypertrophic and bushy appearance in all three TBI + HS groups compared to the Naïve + Sham group. Treatment with 20 ml/kg PNPH treatment modestly reduced the number of hypertrophic and bushy Iba1-positive microglia when compared with LR-treated TBI piglets (*P* = 0.04; Figure 6E).

### Physiological results with TBI alone

**Figure 7:**
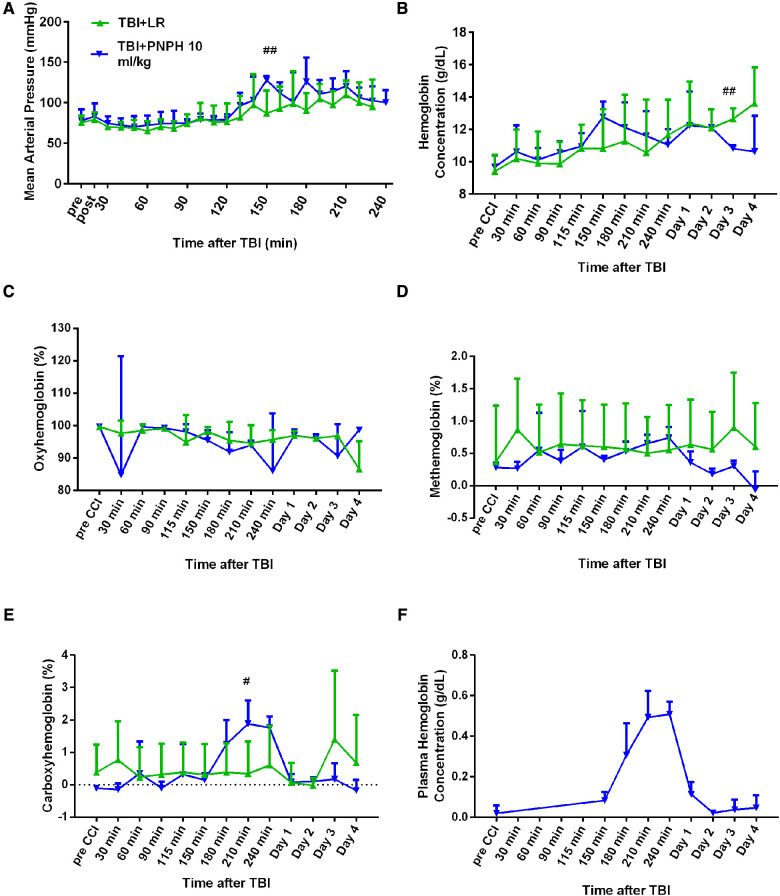
Systemic physiologic changes after TBI without hemorrhagic shock. Mean arterial pressure (**A**), hemoglobin concentration (**B**), percent of oxygenated hemoglobin (**C**), percent of methemoglobin (**D**), and percent of carboxyhemoglobin (**E**) in whole blood for pigs subjected to TBI followed by infusion of 10 ml/kg of lactated Ringer's (LR) solution or PNPH at 120–180 after TBI. Sample sizes per time point were 4–6 for LR group and 5–6 for 10 ml/kg PNPH group (smaller sample sizes occurred at 180–240 min when arterial blood samples could no longer be drawn because the pig was too active or the subcutaneously-routed catheter was inoperable). Individual data are shown in Supplemental Figures 6–10. PNPH infusion produced an increase in plasma hemoglobin concentration (**F**). Values are means ± SD. ^#^*P* < 0.05, ^##^*P* < 0.01 LR infusion vs. PNPH infusion group determined by one-way ANOVA.

The time course of physiologic data is presented for the survivors from each group in the TBI alone experiment in Figure 7, and the individual data are shown at each time point in Supplementary Figures S6–S10. Before TBI, MAP was approximately 80 mmHg and remained stable during the first 2 h after TBI in the absence of hemorrhagic shock in the groups destined to receive 10 ml/kg LR or 10 ml/kg PNPH at 2 h (Figure 7A). Afterwards, MAP gradually increased in both TBI groups as the pigs regained consciousness. Some pigs receiving PNPH had brief episodes of arterial hypotension that were restored by infusion of epinephrine. However, infusion of epinephrine sometimes resulted in an overshoot in MAP, which may have accounted for the elevated MAP seen at 150 min and 180 min time points after TBI. The oncotic effect of PNPH might also be a factor in this early increase in MAP. At the remaining time points, MAP in both TBI groups was similar to that in the sham group.

During the first 4 h after TBI, both groups had similar levels of whole blood Hb (Figure 7B), FHbO2 (Figure 7C), and metHb (Figure 7D). PNPH infusion resulted in the expected increase in whole blood COHb, but the peak level was only 2% COHb and was eliminated by day 1 (Figure 7E). Plasma Hb concentration increased to 0.51 ± 0.06 g/dl after 10 ml/kg PNPH infusion and declined to 0.11 ± 0.06 and 0.02 ± 0.01 g/dl on days 1 and 2, respectively (Figure 7F).

### Neuropathology with TBI alone

**Figure 8:**
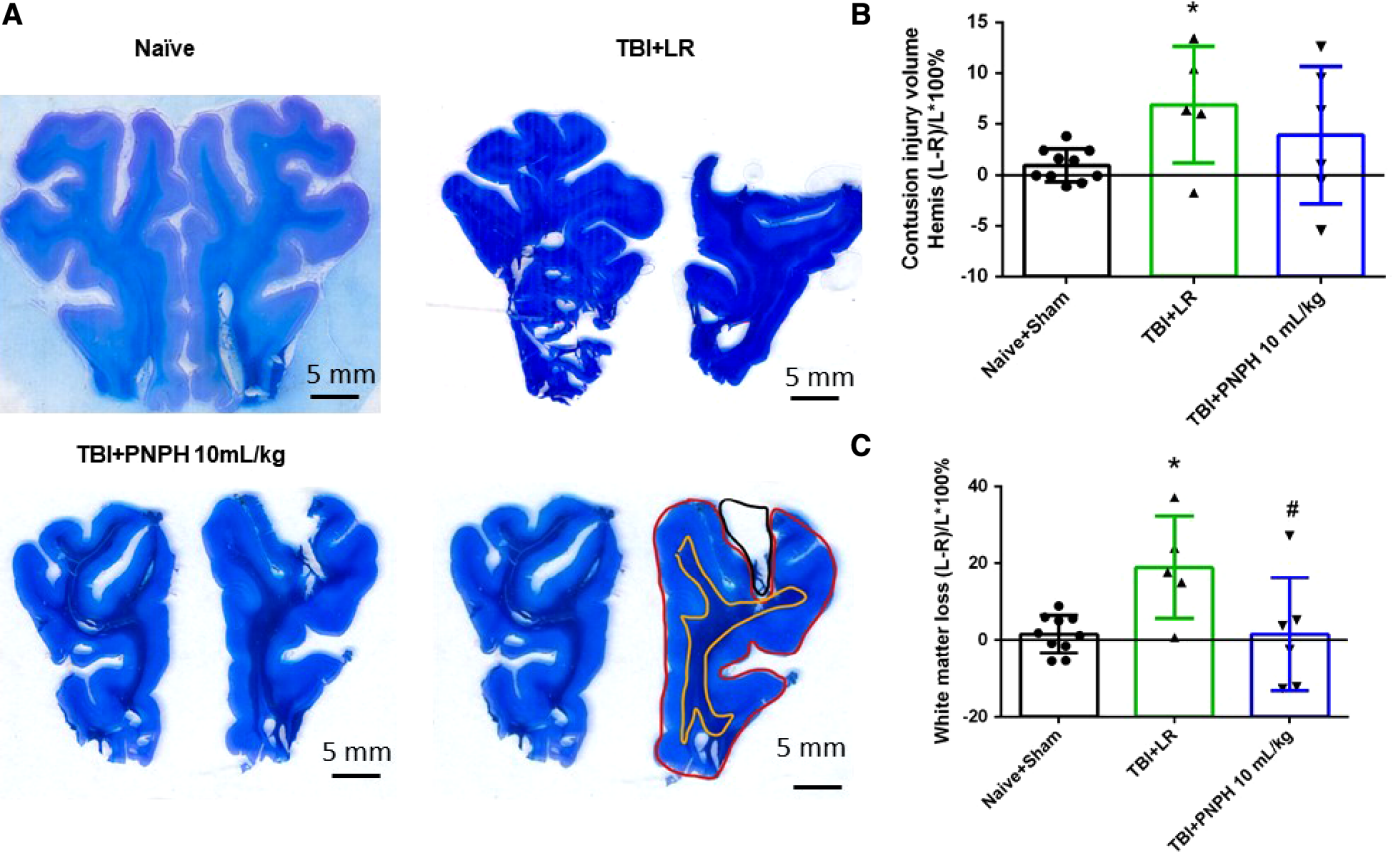
Effect of PNPH on frontal lobe contusion injury volume and volumetric white matter loss after TBI without hemorrhagic shock. (**A**) Representative coronal sections (both hemispheres, top is superior) of frontal lobe of normal pig brain and brains 4 days after TBI and infusion of 10 ml/kg lactated Ringer's (LR) solution and 10 ml/kg PNPH. Gray matter is visualized with cresyl violet staining and subcortical white matter myelin is visualized with Luxol fast blue staining. Area delineated by red trace is the entire histologically normal hemisphere, area delineated by the yellow trace is the white matter, and area delineated by the black trace is the contusion site. (**B**) Contusion injury volume (intact tissue difference of left minus right hemisphere as a percentage of left hemisphere volume contralateral to the injury site) of pigs subjected to TBI followed at 120 min with LR (*n *= 6) or PNPH (*n *= 6). The normal variation in the difference of left minus right hemisphere volume as a percentage of left hemisphere volume is shown for the combined Naïve (*n* = 5) and Sham (*n* = 5) group. (**C**) Volume of white matter loss in the same brains was calculated as the difference between left minus right subcortical white matter volume normalized by the left subcortical white matter volume (contralateral volume). White matter loss was decreased with PNPH treatment. Mean ± SD and individual data for each pig are shown. *P < 0.05 vs. Naïve + Sham group, ^#^*P* < 0.05 vs. LR group determined by one-way ANOVA and the Holm-Sidak procedure for multiple comparisons.

Representative cresyl violet/Luxol fast blue-stained images of coronal brain sections are shown from a naïve pig and pigs with 10 ml/kg LR and 10 ml/kg PNPH infusion started 2 h after TBI (Figure 8A). Contusion lesion volume displayed an overall group effect [*F* (2,19) = 7.513; *P* = 0.004], but there was no significant difference (*P* = 0.17) between the TBI groups receiving LR and PNPH (Figure 8B). The volume of the subcortical white matter on the impacted side relative to the contralateral side displayed an overall group effect [*F* (2,19) = 6.826; *P* = 0.006]. Compared to the Naïve + Sham group, the TBI group receiving LR exhibited a significant loss of white matter volume ipsilateral to the injury (*P* = 0.022) (Figure 8C). In the TBI group receiving PNPH, the loss of white matter volume was significantly attenuated compared to the group receiving LR (*P* = 0.027). The value in the PNPH group did not significantly differ from the value in the Naïve + Sham group (*P* = 0.99).

**Figure 9:**
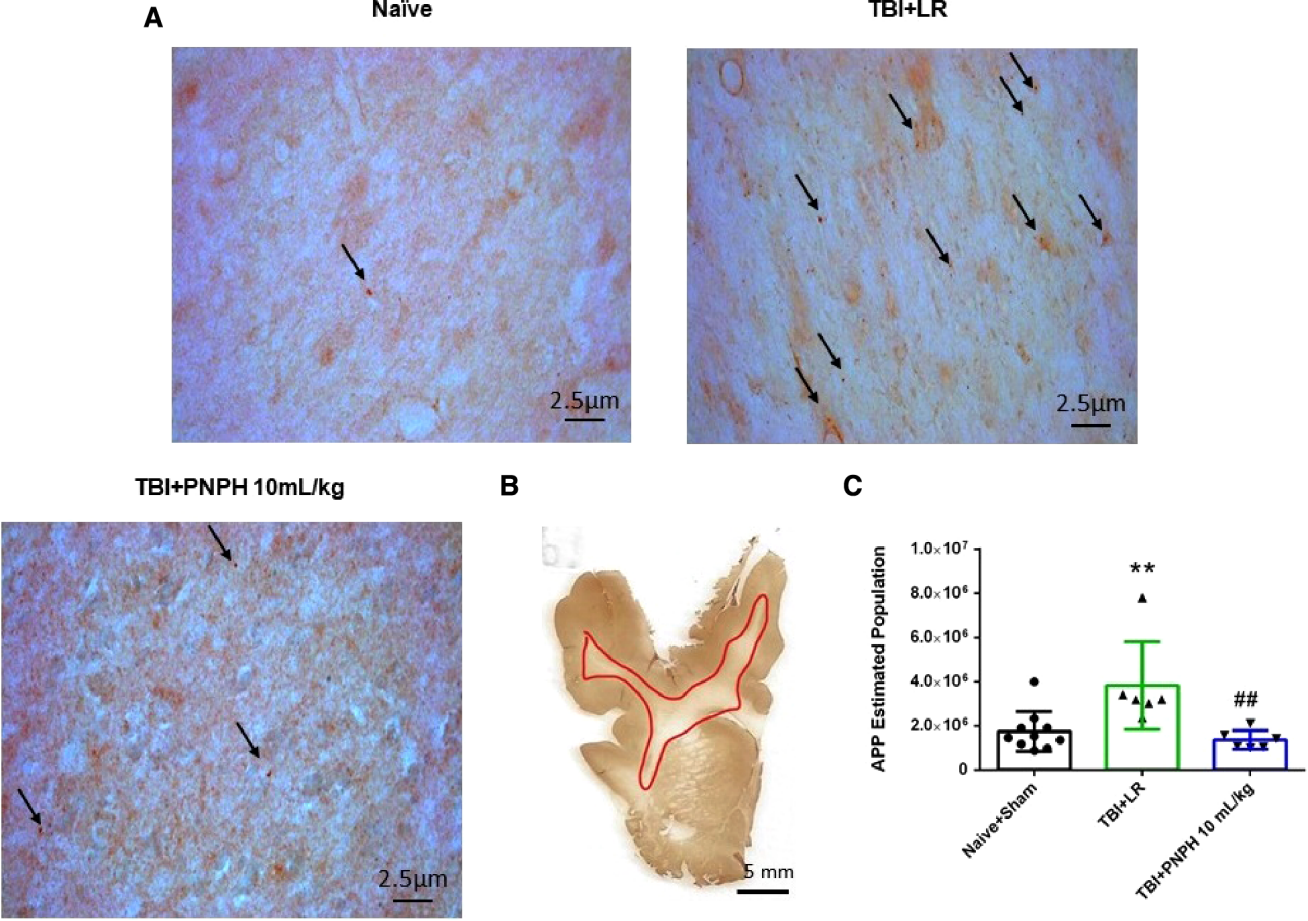
Effect of PNPH on APP expression after TBI without HS. (**A**) Representative images of APP immunostaining from naïve pig and pigs infused with 10 ml/kg LR or 10 ml/kg PNPH at 2 h after TBI. Arrows point to particles of APP accumulation. (**B**) The area of subcortical white used in the analysis is illustrated with the red trace. (**C**) The number of APP particles throughout subcortical white matter was estimated with the optical fractionator method of unbiased stereology and are plotted for the combined Naïve + Sham group (*n* = 10) and for TBI groups infused with 10 ml/kg LR (*n* = 6) and 10 ml/kg PNPH (*n* = 6). APP particles increased after TBI with LR infusion but not after PNPH infusion. Mean ± SD and individual data for each pig are shown. **P < 0.01 vs. combined naïve and sham groups, ^##^*P* < 0.01 vs. LR group determined by one-way ANOVA and the Holm-Sidak procedure for multiple comparisons.

Examples of APP immunoreactive particles are shown in Figure 9A for sections from naïve, TBI + LR, and TBI + PNPH pigs, and the area of subcortical white matter used in the stereological analysis is shown in Figure 9B. The quantification of APP particles in subcortical white matter in the TBI alone experiment (Figure 9C) showed a significant overall effect of group intervention [*F* (2,19) = 7.513; *P* = 0.004]. The Holm-Sidak procedure for multiple comparisons indicated that the estimate of the number of APP particles in the TBI + LR group (3,826,066 ± 1,977,136) was significantly greater than the number in the Naïve + Sham group (1,749,836 ± 908,968, *P* = 0.007). The number in the TBI + LR group was also significantly greater than that in the TBI + PNPH group (1,365,371 ± 429,906, *P* = 0.007). Interestingly, the value in the TBI + PNPH group was not significantly different from that in the Naïve + Sham group. Thus, the benefit of PNPH treatment on both white matter volume and APP accumulation seen in the TBI + HS experiment persisted in the absence of hemorrhagic shock.

**Figure 10:**
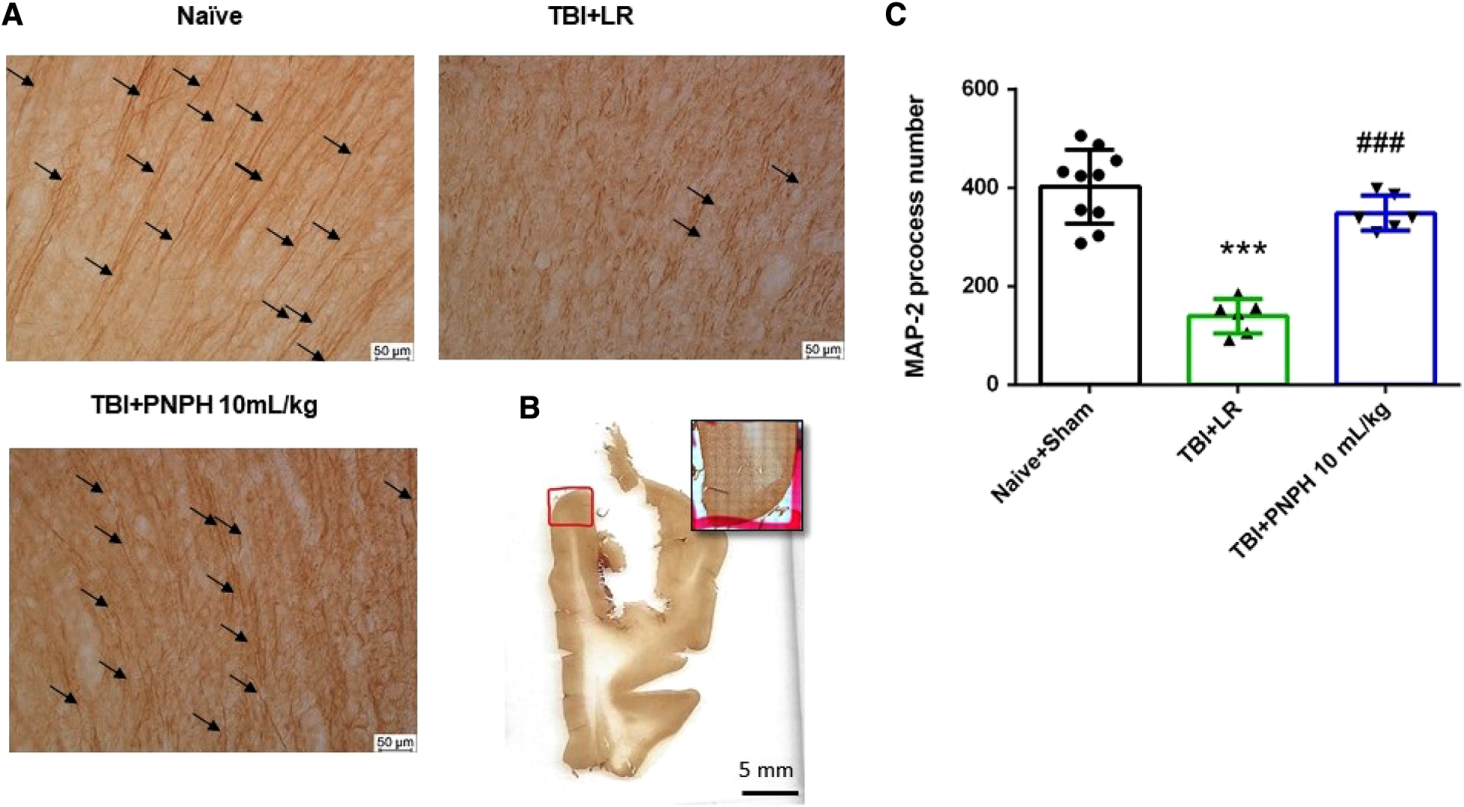
Effect of PNPH on MAP-2–identified gray matter neuronal dendrites after TBI without hemorrhagic shock. (**A**) Representative images of MAP-2 immunostaining from naïve pig and pigs infused with 10 ml/kg LR or 10 ml/kg PNPH 2 h after TBI. Arrows point to dendritic segments with >50 µm length from gyrus of the cortex adjacent to the gyrus where CCI was produced. (**B**) Example of area of gyrus analyzed is outlined in red. (**C**) The number of long neocortical dendrites per coronal section averaged over 10–12 sections are plotted for the combined Naïve + Sham group (*n* = 10) and for TBI groups infused with 10 ml/kg LR (*n* = 6) and 10 ml/kg PNPH (*n* = 6). The number of long dendrites was decreased in the TBI group infused with LR compared to the Naïve + Sham group and to the TBI group infused with PNPH. Mean ± SD and individual data for each pig are shown. ***P < 0.001 vs. combined Naïve + Sham groups, ^###^*P* < 0.001 vs. LR group determined by one-way ANOVA and the Holm-Sidak procedure for multiple comparisons.

Images of MAP-2–positive dendritic segments with >50 µm length in the gyrus adjacent to the contusion are shown in Figure 10A for brains from the TBI alone experiment, and an example of the area of the gyrus used in the analysis is shown in Figure 10B. As occurred with TBI + HS, one-way ANOVA indicated a significant overall effect of group intervention on dendritic integrity [*F* (2,19) = 40.465; *P* < 0.001]. The Holm-Sidak procedure for multiple comparisons revealed a marked decrease in the number of intact dendritic processes with a length >50 µm in the TBI group treated with LR (139 ± 35 per coronal section; *P* < 0.001) compared to the Naive + Sham group (403 ± 75 per coronal section) (Figure 10C). Treatment with PNPH greatly attenuated this decrease in long dendrites (349 ± 36 per coronal section; *P* < 0.001 from LR group), and the values were only marginally different from the Naïve + Sham group (*P* = 0.09). Again, the benefit of PNPH treatment after TBI did not require the presence of HS.

**Figure 11:**
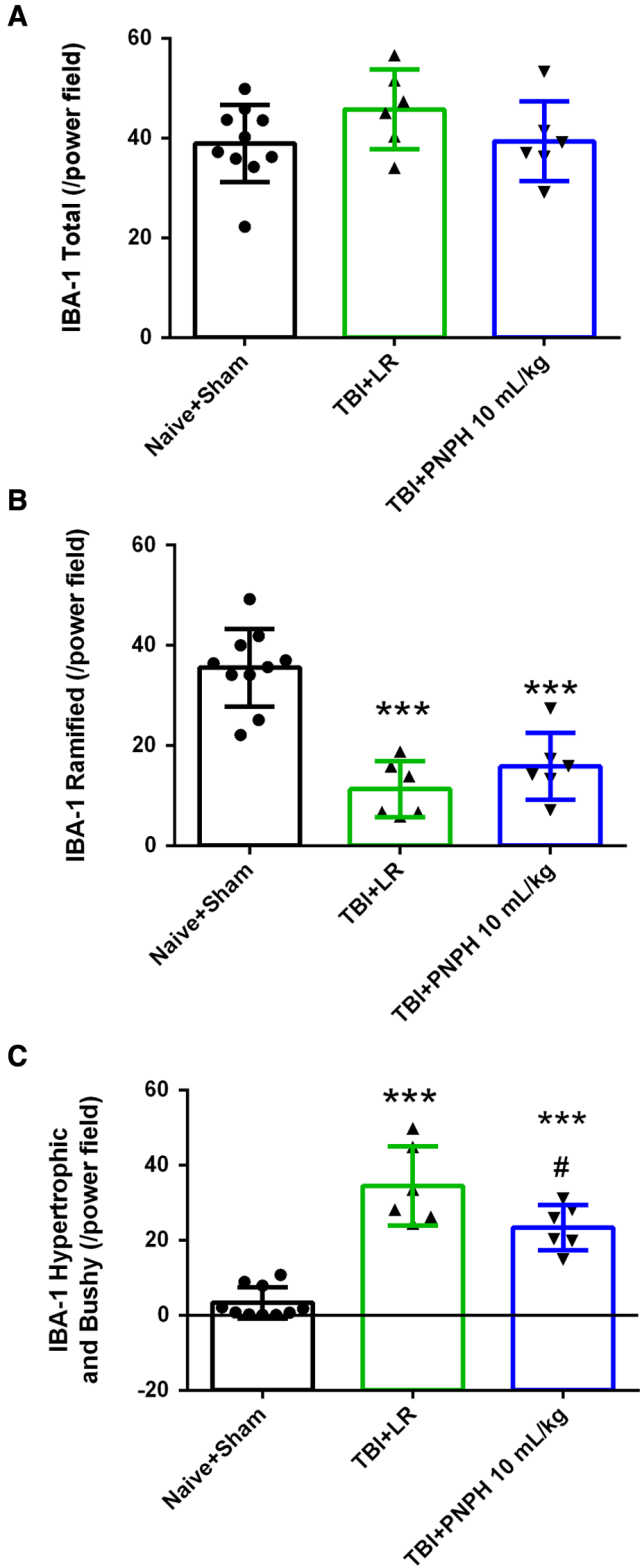
Effect of PNPH on microglial activation after TBI without HS. The number of Iba-1–positive cells (**A**), ramified Iba-1–positive cells (**B**), and hypertrophic plus bushy Iba-1–positive cells (**C**) are plotted for the combined Naïve + Sham group (*n* = 10) and for TBI groups infused with 10 ml/kg lactated Ringer's (LR) solution (*n* = 6) and 10 ml/kg PNPH (*n* = 6). The number of Iba-1–positive cells did not change significantly 4 days after TBI, whereas the number of Iba-1 cells classified as ramified decreased significantly in both TBI groups. Hypertrophic and bushy Iba-1 cell number markedly increased in the TBI group with LR infusion and the increase was attenuated with PNPH infusion. Mean ± SD and individual data for each pig are shown. ***P < 0.001 vs. combined Naïve + Sham group, ^#^*P *< 0.05 vs. TBI group with LR treatment determined by one-way ANOVA and the Holm-Sidak procedure for multiple comparisons.

With TBI alone, the density of Iba-1–positive cell counts showed no overall effect among the three groups [*F* (2,19) = 1.620; *P* = 0.22] (Figure 11A). Analysis of the number of Iba-1–positive cells classified as having ramified processes yielded an overall group effect [*F* (2,19) = 27.810; *P* < 0.001] accompanied by significant decreases in the LR and PNPH groups compared to the Naïve + Sham group (Figure 11B). The difference between the LR and PNPH groups was not significant. On the other hand, the number of hypertrophic/bushy Iba-1–positive cells at 4 days after TBI alone displayed an overall group effect [*F* (2,19) = 42.107; *P* < 0.001] in which the significant increase seen with LR infusion (*P* < 0.001) compared to the Naive + Sham group was significantly attenuated with PNPH treatment (*P* = 0.011 vs. LR group).

## Discussion

The CCI model of TBI has been used in infant and prepubertal juvenile pigs, but many studies had short survival times of 6 h or less (38, 47) and the number of studies with neuropathologic analysis at longer survival time is limited (34, 48, 49). Unique features in the present study include the immunohistochemical analysis of cellular changes in neocortical regions adjacent to the impacted gyrus. We purposely performed the impact over the dorsolateral frontal lobe region to avoid direct injury to primary somatosensory and motor neocortices that could adversely affect the pig's mobility and ability to eat and drink. Also noteworthy is that the pigs were approximately 3-months old, an age that approximates the age range that often appears in the literature for pre-pubertal swine models of TBI + HS (35, 38–42). The weight of the brain at this age is approximately 75% of those in fully mature pigs (50). Thus, the brains are likely to have undergoing myelination and brain development, which may have influenced neuronal vulnerability.

Our first main finding is that resuscitation from TBI + HS with 10 ml/kg and 20 ml/kg PNPH at 2 h after TBI substantially ameliorated the overall loss of white matter and neuronal cellular injury in the gyrus adjacent to the impact. The cerebroprotection was associated with an improvement in gray and white matter and an attenuation of neuroinflammation as assessed by morphologic changes of microglia. Specifically, PHPH protected neocortical neuron dendrites as seen by MAP-2 immunoreactivity, diminished axonopathy as revealed by APP immunoreactivity, and rescued myelination perturbation as seen by the Luxol fast blue staining. The second main finding was that similar benefits were observed when PNPH was infused 2 h after TBI without intervening HS, thereby implying that hemorrhagic hypotension is not a requirement for cerebroprotection by PNPH in this large animal model of TBI and that the cerebroprotection is not completely dependent on a faster restoration of MAP by PNPH infusion. This protection is presumably attributable to nitroxide-based scavenging of superoxide and peroxynitrite within the vascular space, resulting in less vascular damage and inflammation, and possibly in the extravascular space where there is blood-brain barrier disruption (23, 24). Moreover, PNPH serves as a CO-releasing molecule that likely reinforces the anti-inflammatory effects exerted by nitroxide free radical scavenging (20) and that can promote cerebral vasodilation, even in the absence of nitroxide conjugation to PEG-Hb (51, 52). Furthermore, the ability of plasma-based PNPH to enter compressed capillaries that restrict red blood cell entry and to reduce edema (12, 13) may also improve microcirculatory hemodynamics independent of changes in MAP.

We analyzed the volumetric change of intact tissue after CCI and found that although the contusion injury volume in cerebral cortex was, on average, smaller in the groups treated with PNPH, the variability was relatively large and the difference from the LR-treated group did not attain statistical significance in either the TBI + HS experiment or the TBI alone experiment. Variability in contusion volume in the CCI model is not unusual. The coefficient of variation of contusion volume in the TBI + HS + LR group obtained histologically on Day 4 was 48% (SD = 6.11%; mean = 12.60%) and is comparable to that reported by others (42) in a TBI + HS swine model with T2 MRI measurements of edema on Day 3 after CCI (coefficient of variation of 46%, SD = 551 mm^3^, mean = 1,202 mm^3^). In rodent CCI models, variability has been attributed to several factors related to the instrumentation, experimental protocol, and presence of hemorrhage (53, 54). For CCI in a gyrencephalic brain, there are many complex considerations such as whether the center of the impact approximates the center of the underlying gyrus or is over a sulcus and whether the trajectory of the subcortical white matter is parallel or perpendicular to the impact force. It should also be noted that contusion injury volume may continue to expand beyond 4 days, and a treatment effect might have become more evident at a later time point.

White matter volume was measured by staining of myelin with Luxol fast blue on sections in the frontal lobe. The 26% decrease in subcortical white matter volume relative to the contralateral white matter volume seen in the TBI + HS + LR group was substantially attenuated with PNPH resuscitation. In the case of TBI alone, PNPH almost completely prevented white matter loss. To specifically assess axonopathy, we measured accumulation of APP particles, which are known to accumulate after disruption of axonal transport induced by TBI (55). Moreover, TBI is considered a risk factor for Alzheimer's disease, either as a precipitating event or by accelerating development of the disease (56). The accumulation of APP after TBI and increased amyloidogenic (Aβ-producing) processing of APP by β-secretase has been postulated as a major cause of brain accumulation of Aβ (57, 58). TBI produced an increase the number of APP-positive white matter inclusions (brown dots) in both the TBI + HS and TBI alone experiments. Remarkably, treatment with PNPH largely prevented the increase in APP particle accumulation after TBI + HS and TBI alone. Together with the results of white matter volumetric improvement, PNPH appears to mitigate markers of axonopathy and myelin loss.

MAP-2 is a neuron-specific cytoskeletal protein that stabilizes microtubules in dendrites and the cell body by linking with intermediate filaments and other microtubules. Because MAP-2 is found along the length of the microtubules that are enriched in neuron dendrites, it is a reliable marker of dendrite integrity. We found significant neocortical dendritic disruption, as indicated by a decrease in the length of continuous MAP-2 stained dendrites within the plane of the tissue sections. The decrease in the number of dendrites with a length greater than 50 µm was measured in cerebral cortex independent of layer throughout the gyrus adjacent to the gyrus that was impacted and thus is indicative on neuronal injury remote from the contusion. This decrease occurred in the LR groups with and without concomitant HS and thus is primarily related to the CCI itself. Importantly, treatment with PNPH ameliorated the decrease in the number of long dendrites in gray matter. This observation indicates that cytoskeletal disruption in gray matter remote from the mechanically induced contusion is a treatable phenomenon, even when the treatment is delayed by 2 h after the primary insult. Therefore, delayed PNPH infusion has the capability of rescuing not only axonopathy, but also dendrites.

Neuroinflammation after TBI is usually accompanied by morphologic changes in microglia consisting of increased size of the cell body, less ramification of fine processes giving rise to shorter, hypertrophic processes, and in some cases, severe shortening and thickening of processes around the cell body that convey a bushy appearance (44, 48). As expected, pig brains in the TBI + HS and TBI alone protocols presented with these characteristic changes. Interestingly, treatment with PNPH in both protocols attenuated the increase in the number of microglia with the hypertrophy and bushy appearance without affecting the decrease in microglia with fine process ramification. This pattern suggests that microglia are still activated but that not as many are in an overt pro-inflammatory state. Measurements of cytokines would be needed to help confirm this interpretation. In addition, the number of positive Iba1-positive cells significantly increased at 4 days after TBI + HS with LR resuscitation, and this increase was significantly reduced by treatment with 20 ml/kg PNPH. Although some of the increase may be attributable to infiltrating macrophages, it seems likely that PNPH also limited microglia proliferation. The latter would also be consistent with an anti-inflammatory effect of PNPH.

Previous studies with PNPH resuscitation from TBI + HS in mouse (12, 13, 26) and guinea pig (31) demonstrated faster recovery of MAP than with LR. Here, we did not observe substantial differences in the rate of MAP recovery after TBI + HS among groups, most likely because we used a faster rate of LR infusion to achieve a greater total treatment volume with LR (twice the hemorrhage volume) than with PNPH over a similar amount of time. Moreover, the infusion time was prolonged so as not to produce rapid changes in preload or afterload on the heart.

Infusion of 10 and 20 ml/kg PNPH produced dose-dependent increases in the plasma concentration of this cell-free Hb. Measurable levels were detected at 1 day after 10 ml/kg infusion and at 2 days after 20 ml/kg infusion. These recovery levels are consistent with a circulating half-life in the range of 12–24 h that is dependent on the amount of infused PNPH (zero-order clearance kinetics) in this large animal model. The peak concentration of Hb in the plasma remained less than 1 g/dl. PNPH resuscitation after TBI + HS limited the decrease in total blood Hb concentration seen with LR resuscitation. The concentration of Hb in the plasma may have contributed to attenuating the decrease in total Hb, but the larger volume of LR infused at a faster rate than PNPH is probably another factor that needs to be considered in the more profound hemodilution observed in the TBI + HS + LR group.

One concern with the use of cell-free Hb is that metHb can increase and produce oxidative stress. However, the percent of metHb in whole blood increased by only 1% of all Hb at 240 min after TBI + HS and subsided by one day. No significant elevations of metHb were observed with PNPH infusion in the TBI alone group. Thus, adverse effects on metHb levels are minimal with PNPH infusion, likely because the nitroxide groups on the molecule limit autooxidation (22, 23). Moreover, any adverse effects of this mild increase in metHb is expected to be alleviated by the peroxidase activity of nitroxides limiting the transition of the heme iron from the ferric to the highly reactive ferryl state (28, 29).

PNPH is stored as COHb to prevent autooxidation before it is infused. Upon infusion, CO is released and exchanges with Hb in red blood cells. The level of COHb increased to approximately 2% after infusion of 10 ml/kg PNPH and 4% after infusion of 20 ml/kg PNPH. This reservoir of CO storage may provide anti-inflammatory effects for the early hours after TBI until the CO is excreted by the lungs (20, 21). Importantly, these low levels of COHb did not have a significant effect on the fraction of arterial Hb carrying oxygen.

Although PNPH treatment provided significant cerebroprotection in 4-day survivors in both the TBI + HS and TBI alone models, a major limitation was that PNPH also produced severe hypotension in many of the pigs and required infusion of epinephrine to maintain MAP above 40 mmHg in many of the pigs. In some cases, the infusion of epinephrine was inadequate to prevent cardiac arrest. When the hypotension occurred, it was usually rapid. In some pigs, the MAP began to drop as early as 2 min into the infusion when the pig received only 5 ml of PNPH, which is a small volume relative to the >2 L blood volume of a 28-kg pig. This effect was unexpected because hypotension was not observed in mouse or guinea pig during PNPH infusion. The solvent for PNPH was dialyzed extensively against LR to remove all free reagents that could affect cardiac function and cause hypotension. One consideration is that pseudo-anaphylactic reaction to the PEG itself, although infrequent (59), might become more probable by changes in the immune milieu 2 h after TBI in 3-month-old swine. Left ventricular function has been reported to be depressed over the early hours after TBI in the absence of HS in 26-kg pigs (60), comparable to the size pig we used, and may provide the conditions for increased sensitivity to an anaphylactic stimulus. The use of midazolam to provide sedation after TBI may have also suppressed reflex sympathetic activity. Further work is needed to identify the underlying mechanism before recommending PNPH for use in non-human primates and humans.

## Conclusion

Collectively, the presented data on myelin staining, APP particle number, dendrite length and microglial activation indicate that resuscitation from TBI with PNPH is superior in survivors in protecting the peri-contusion brain tissue compared to resuscitation with LR. These results extend previous work in rodent models of TBI + HS and demonstrate that PNPH possesses the capability to protect various aspects of neuronal integrity in the large gyrencephalic brain of the pig. This protection persisted when PNPH infusion was delayed by 2 h after TBI in the absence of HS. However, adverse effects on cardiac function and hemodynamics that appear to be specific to swine need to be further investigated before translation to human use.

## Data Availability

The raw data supporting the conclusions of this article will be made available by the authors, without undue reservation.
