# Supplementary material for: Polynitroxylated PEGylated hemoglobin protects pig brain neocortical gray and white matter after traumatic brain injury and hemorrhagic shock
Source: Front Med Technol. 2023 Feb 21;5:1074643. doi: 10.3389/fmedt.2023.1074643 (PMC9988926; doi:10.3389/fmedt.2023.1074643)
Supplement: Supplementary file 1 [file Datasheet1.pdf]

Supplemental Figures

Polynitroxylated PEGylated hemoglobin protects pig brain neocortical gray and white matter after traumatic brain injury and hemorrhagic shock

Jun Wang[1], Yanrong Shi[1], Suyi Cao[1], Xiuyun Liu[1], Lee J. Martin[2], Jan Simoni[3], Bohdan J. Soltys[3], Carleton J.C. Hsia[3], Raymond C. Koehler[1]

1. Department of Anesthesiology and Critical Care Medicine, Johns Hopkins University, Baltimore, MD, USA
2. Department of Pathology, Johns Hopkins University, Baltimore, MD, USA
3. AntiRadical Therapeutics LLC, Sioux Falls, SD, USA

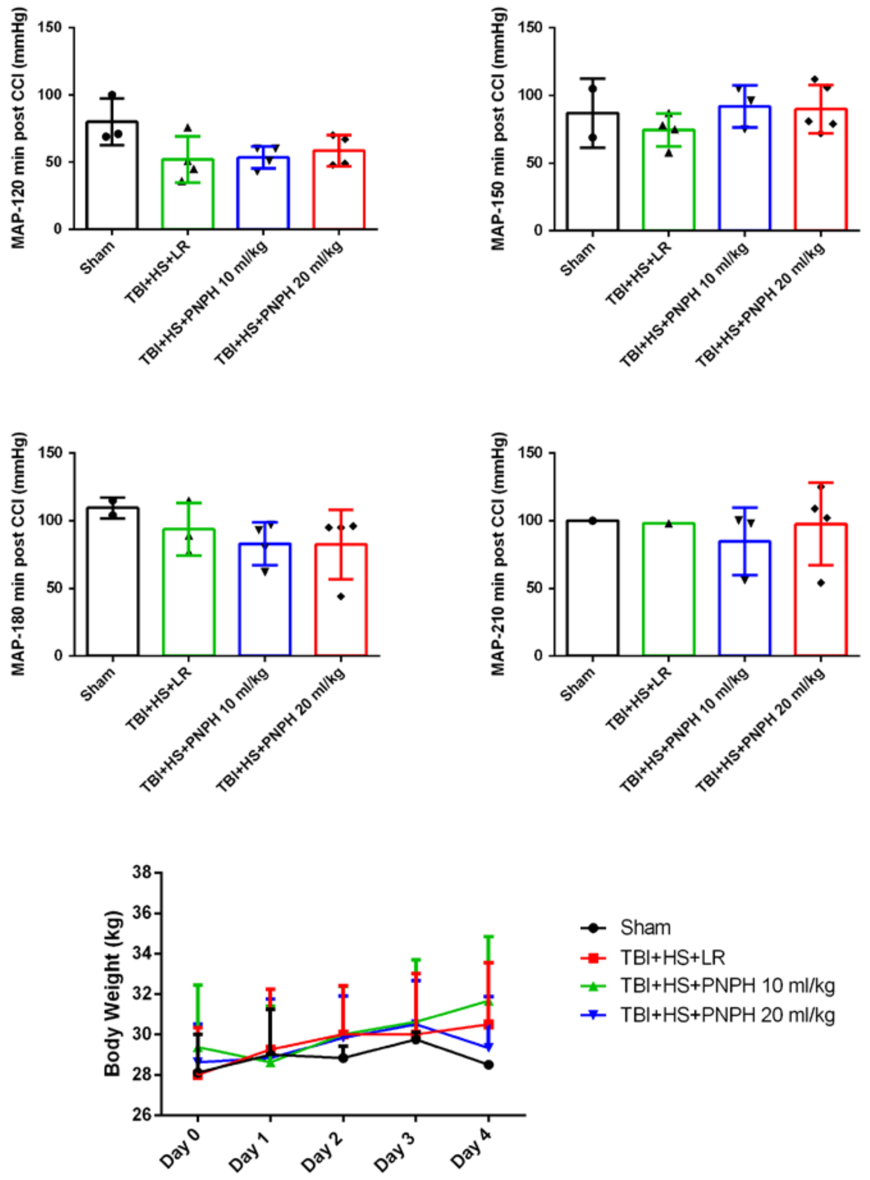

Supplemental Figure 1. Individual data and mean ±SD of mean arterial blood pressure (MAP) in the TBI+hemorrhagic shock (HS) experiment at 120, 150, 180 and 210 min after TBI in groups resuscitated with 60 ml/kg of lactated Ringer's solution (LR) or 5, 10, or 20 ml/kg of PNPH (infusion starting at 120 minutes after TBI) or at equivalent times in a Sham surgery group. There were no significant differences in MAP during resuscitation among groups by one-way ANOVA. For example, at 120 min, $F(3,11) = 2.865$, $P = 0.085$ for MAP with $n = 3$ Shams, 5 for LR group, 4 for 10 ml/kg PNPH group, and 5 for 20 ml/kg PNPH group. Note that 2 arterial catheters stopped working by 120 min in the Sham group resulting in a small sample size, and that the sample sizes decreased after 120 min in all groups as pigs became ambulatory and too active to obtain valid readings or the subcutaneously-routed catheter became inoperable. Bottom graph shows the body weight before surgery (Day 0) and the 4 post-operative days with n = 5 for Shams, 5 for LR group, 4 for 10 ml/kg PNPH group, and 5 for 20 ml/kg PNPH group.

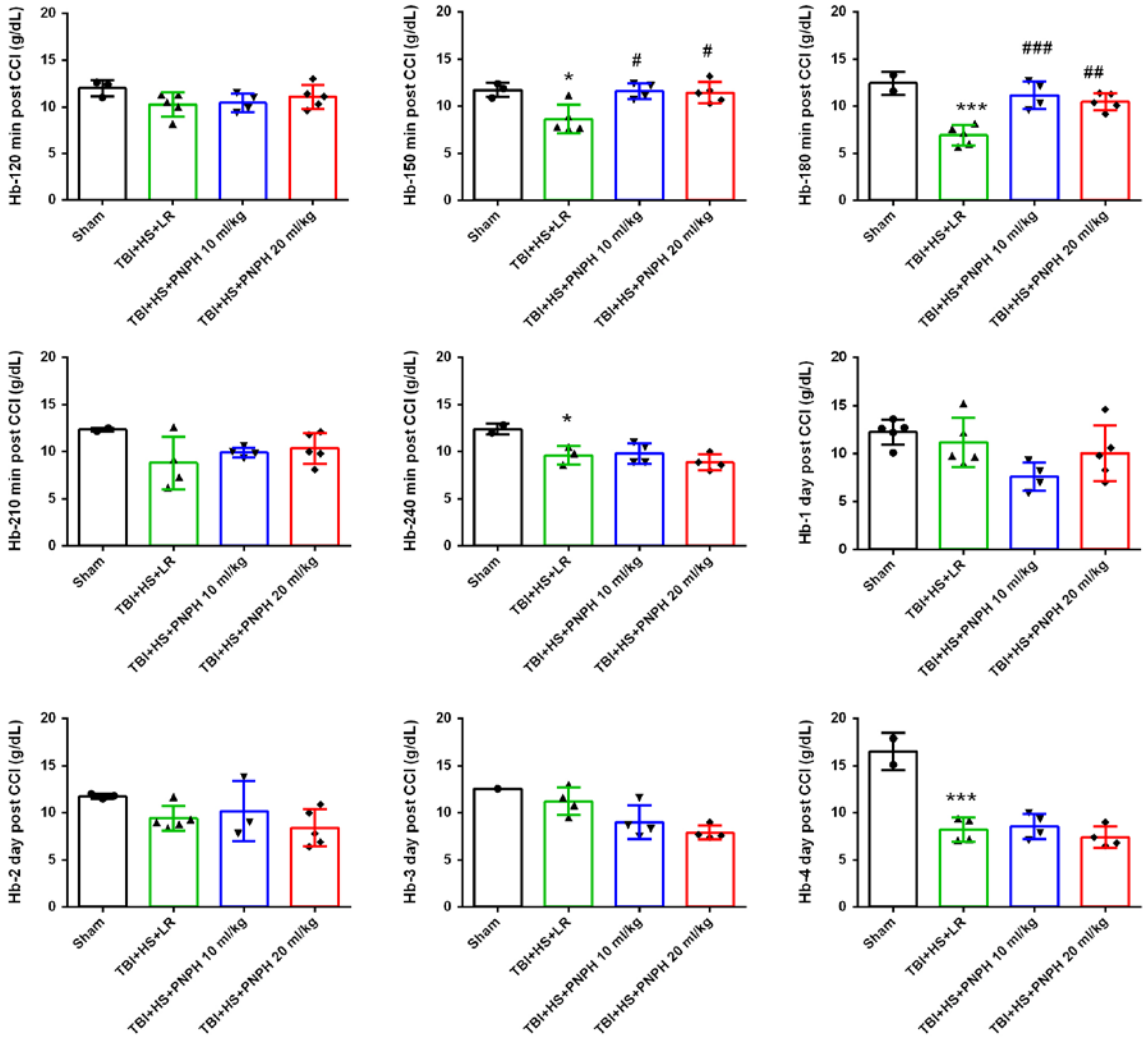

Supplemental Figure 2. Individual data and mean ±SD of blood hemoglobin (Hb) concentration (g/100 ml) in the TBI+hemorrhagic shock (HS) experiment at 120, 150, 180, 210 and 240 minutes and at 1, 2, 3, and 4 days after TBI in groups resuscitated with 60 ml/kg of lactated Ringer's solution (LR) or 5, 10, or 20 ml/kg of PNPH (infusion started at 120 minutes after TBI) and at equivalent times in a Sham surgery group. Data analyzed with one-way ANOVA and the Holm-Sidak procedure: * *P* <0.05, *** *P* <0.001 versus sham group; # *P* <0.05, ## *P* <0.01, ### *P* <0.001 versus LR group; *n* = 3, 5, 4, and 5 for Shams, LR, 10 ml/kg PNPH, and 20 ml/kg PNPH groups, respectively, at 120 min. Note that 2 arterial catheters stopped working by 120 min in the Sham group resulting in a small sample size, and that the sample sizes decreased after 120 min in all groups as pigs became ambulatory and too active to obtain valid readings or the subcutaneous catheter kinked. In some cases, the venous Hb concentration was used at Days 1–4.

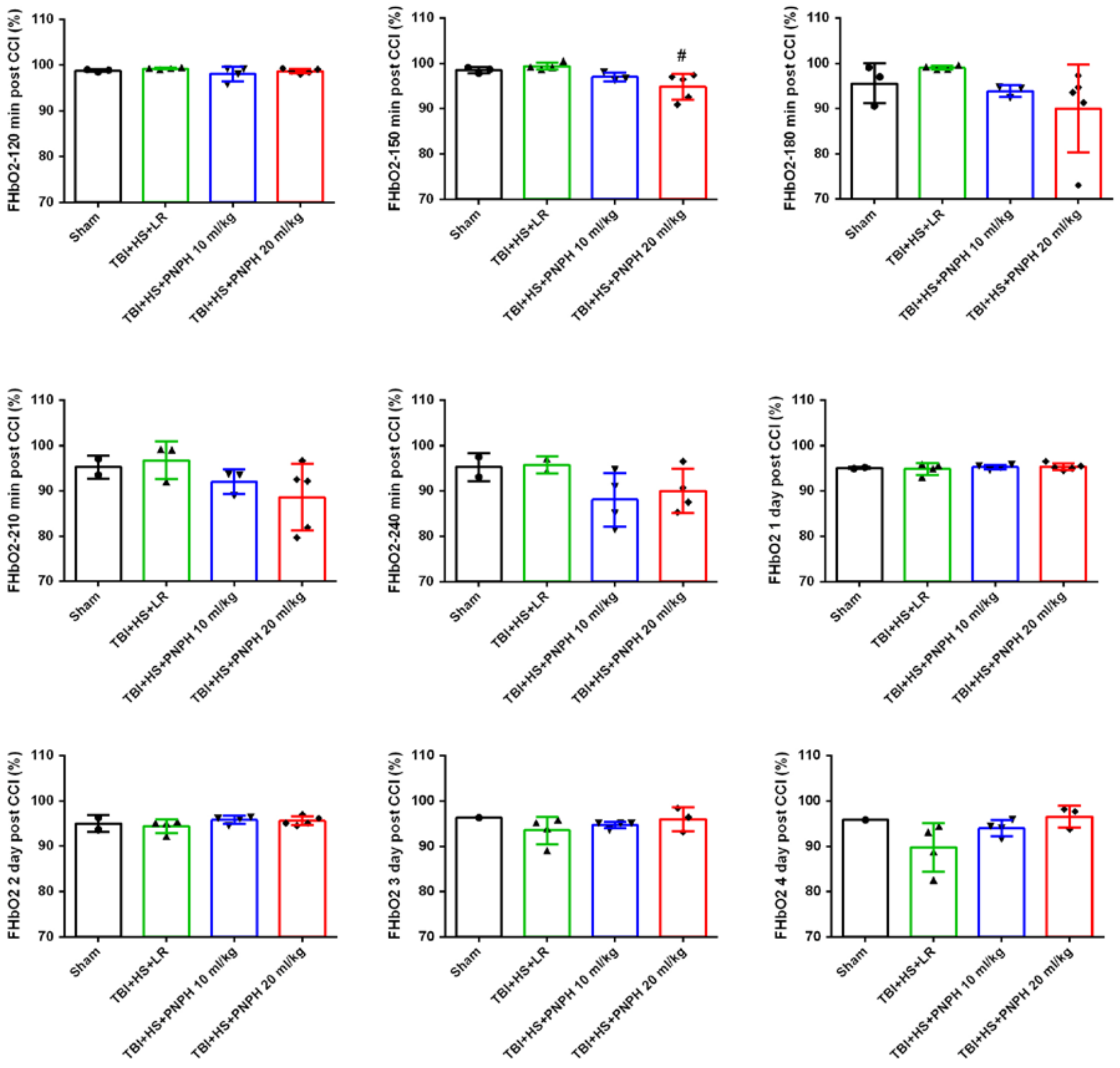

Supplemental Figure 3. Individual data and mean ±SD of the percent of arterial blood hemoglobin carrying oxygen (FHbO2) in the TBI+hemorrhagic shock (HS) experiment at 120, 150, 180, 210 and 240 minutes and at 1, 2, 3, and 4 days after TBI in groups resuscitated with 60 ml/kg of lactated Ringer's solution (LR) or 5, 10, or 20 ml/kg of PNPH (infusion started at 120 minutes after TBI) and at equivalent times in a Sham surgery group. Data analyzed with one-way ANOVA and the Holm-Sidak procedure: # P <0.05 versus LR group; n = 3, 5, 4, and 5 for Shams, LR, 10 ml/kg PNPH, and 20 ml/kg PNPH groups, respectively, at 120 min. Note that 2 arterial catheters stopped working by 120 min in the Sham group resulting in a small sample size, and that the sample sizes decreased after 120 min in all groups as pigs became ambulatory and too active to obtain valid readings or the subcutaneously-routed catheter became inoperable.

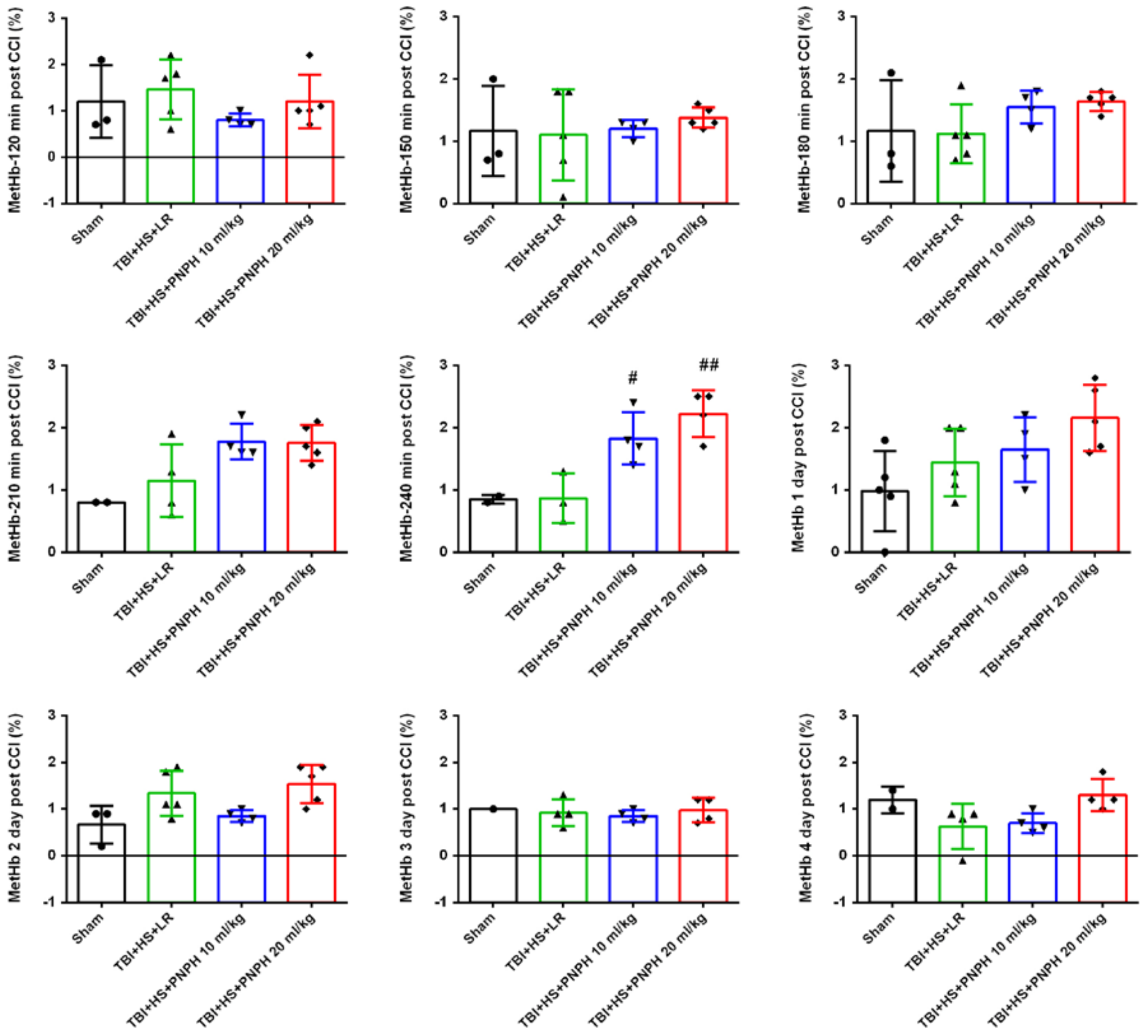

Supplemental Figure 4 Individual data and mean ±SD of the percent of blood methemoglobin (metHb) in the TBI+hemorrhagic shock (HS) experiment at 120, 150, 180, 210 and 240 minutes and at 1, 2, 3, and 4 days after TBI in groups resuscitated with 60 ml/kg of lactated Ringer's solution (LR) or 5, 10, or 20 ml/kg of PNPH (infusion started at 120 minutes after TBI) and at equivalent times in a Sham surgery group. Data analyzed with one-way ANOVA and the Holm-Sidak procedure: # *P* <0.05, ## *P* <0.01 versus LR group; *n* = 3, 5, 4, and 5 for Shams, LR, 10 ml/kg PNPH, and 20 ml/kg PNPH groups, respectively, at 120 min. Note that 2 arterial catheters stopped working by 120 min in the Sham group resulting in a small sample size, and that the sample sizes decreased after 120 min in all groups as pigs became ambulatory and too active to obtain valid readings or the subcutaneously-routed catheter became inoperable. In some cases, the venous Hb concentration was used at Days 1–4.

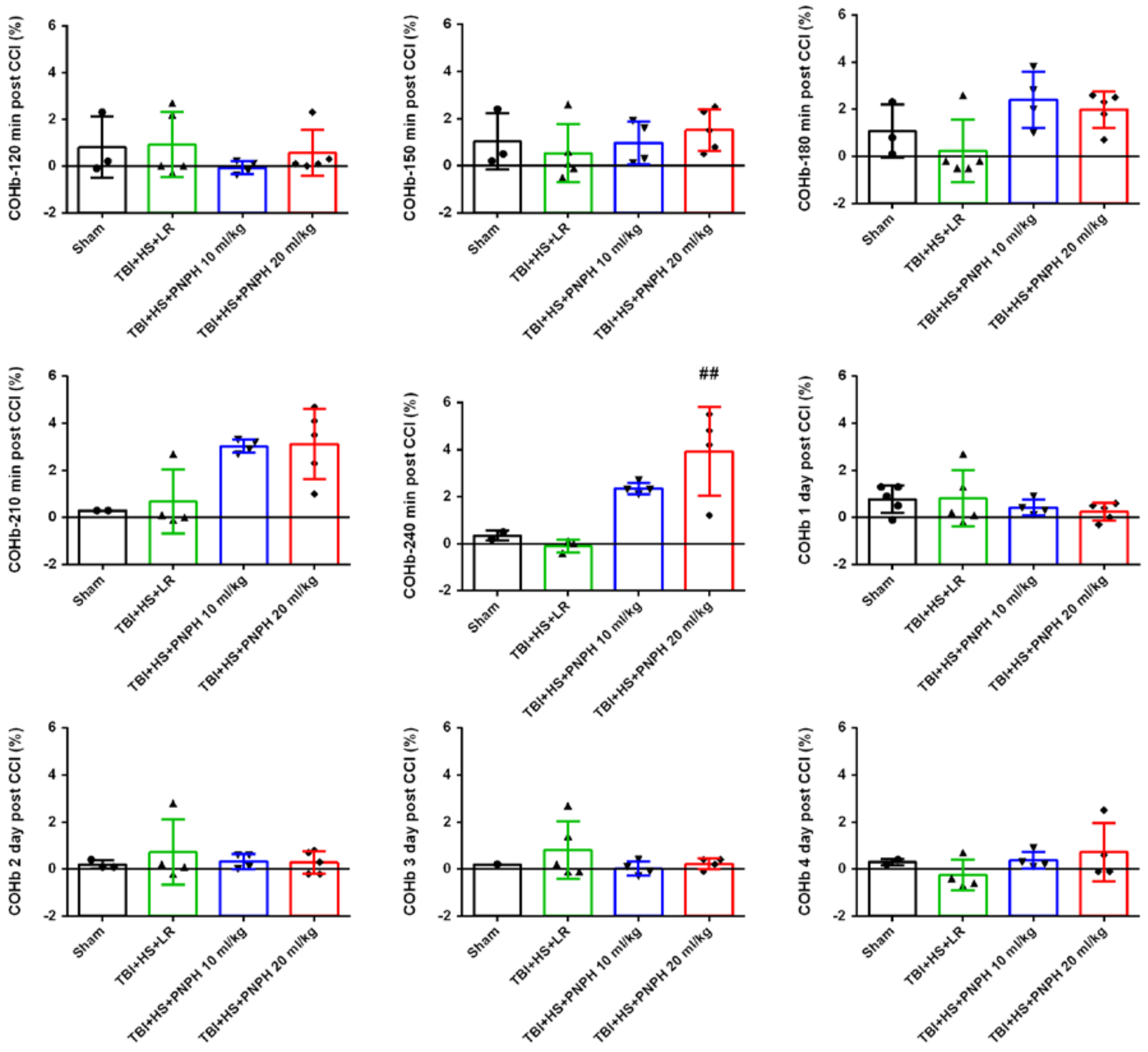

Supplemental Figure 5. Individual data and mean ±SD of the percent of whole blood carboxyhemoglobin (COHb) in the TBI+hemorrhagic shock (HS) experiment at 120, 150, 180, 210 and 240 minutes and at 1, 2, 3, and 4 days after TBI in groups resuscitated with 60 ml/kg of lactated Ringer's solution (LR) or 5, 10, or 20 ml/kg of PNPH (infusion started at 120 minutes after TBI) and at equivalent times in a Sham surgery group. Data analyzed with one-way ANOVA and the Holm-Sidak procedure: # P <0.05, ## P <0.01 versus LR group; n = 3, 5, 4, and 5 for Shams, LR, 10 ml/kg PNPH, and 20 ml/kg PNPH groups, respectively, at 120 min. Note that 2 arterial catheters stopped working by 120 min in the Sham group resulting in a small sample size, and that the sample sizes decreased after 120 min in all groups as pigs became ambulatory and too active to obtain valid readings or the subcutaneously-routed catheter became inoperable. In some cases, the venous Hb concentration was used at Days 1–4.

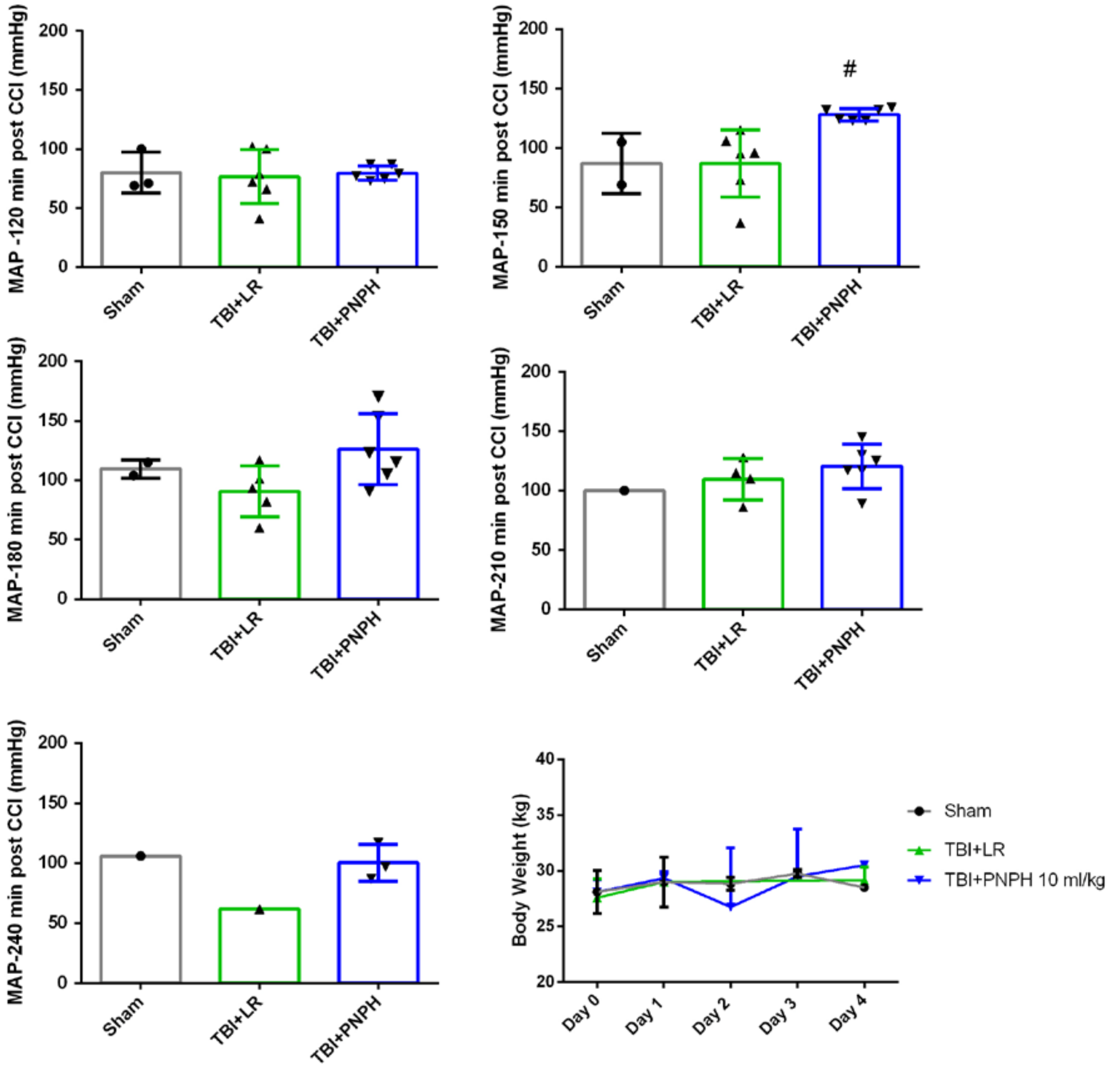

Supplemental Figure 6. Individual data and mean ±SD of mean arterial blood pressure (MABP) in the TBI alone experiment at 120, 150, 180, 210 and 240 minutes after TBI in groups resuscitated with 10 ml/kg of lactated Ringer's solution (LR) or 10 ml/kg of PNPH (infusion started at 120 minutes after TBI) and at equivalent times in a Sham surgery group. Daily body weight mean ±SD shown in bottom right graph. Data analyzed with one-way ANOVA and the Holm-Sidak procedure: # *P* <0.05 versus LR group. ; *n* = 3, 6, and 6 for Shams, LR, 10 ml/kg LR, and 10 ml/kg PNPH groups, respectively, at 120 min. Note that 2 arterial catheters stopped working by 120 min in the Sham group resulting in a small sample size, and that the sample sizes decreased after 120 min in all groups as pigs became ambulatory and too active to obtain valid readings or the subcutaneously-routed catheter became inoperable. In some cases, the venous Hb concentration was used at Days 1–4.

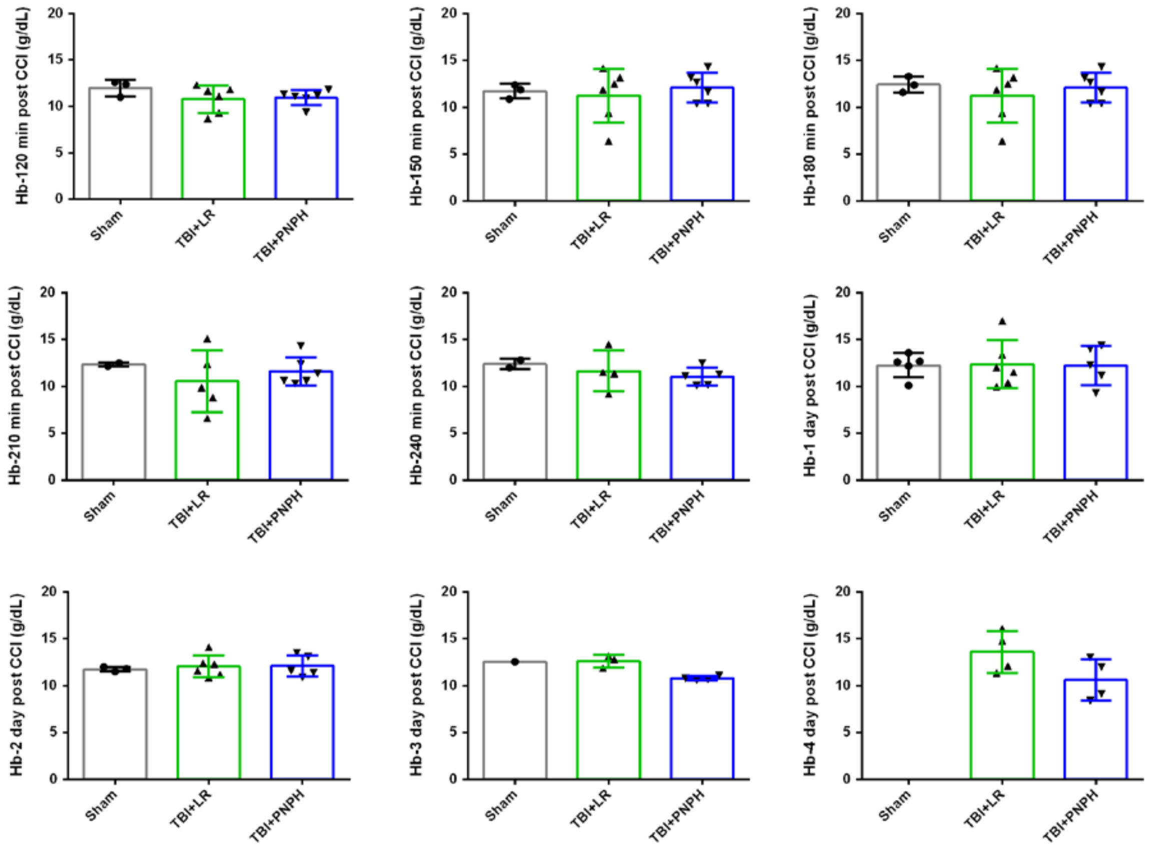

Supplemental Figure 7. Individual data and mean ±SD of whole blood hemoglobin (Hb) concentration (g/100 ml) in the TBI alone experiment at 120, 150, 180, 210 and 240 minutes and at 1, 2, 3, and 4 days after TBI in groups resuscitated with 10 ml/kg of lactated Ringer's solution (LR) or 10 ml/kg of PNPH (infusion started at 120 minutes after TBI) and at equivalent times in a Sham surgery group. There were no significant differences among groups by one-way ANOVA at any time point; *n* = 3, 6, and 6 for Shams, LR, 10 ml/kg LR, and 10 ml/kg PNPH groups, respectively, at 120 min. Note that 2 arterial catheters stopped working by 120 min in the Sham group resulting in a small sample size, and that the sample sizes decreased after 120 min in all groups as pigs became ambulatory and too active to obtain valid readings or the subcutaneously-routed catheter became inoperable. In some cases, the venous Hb concentration was used at Days 1–4.

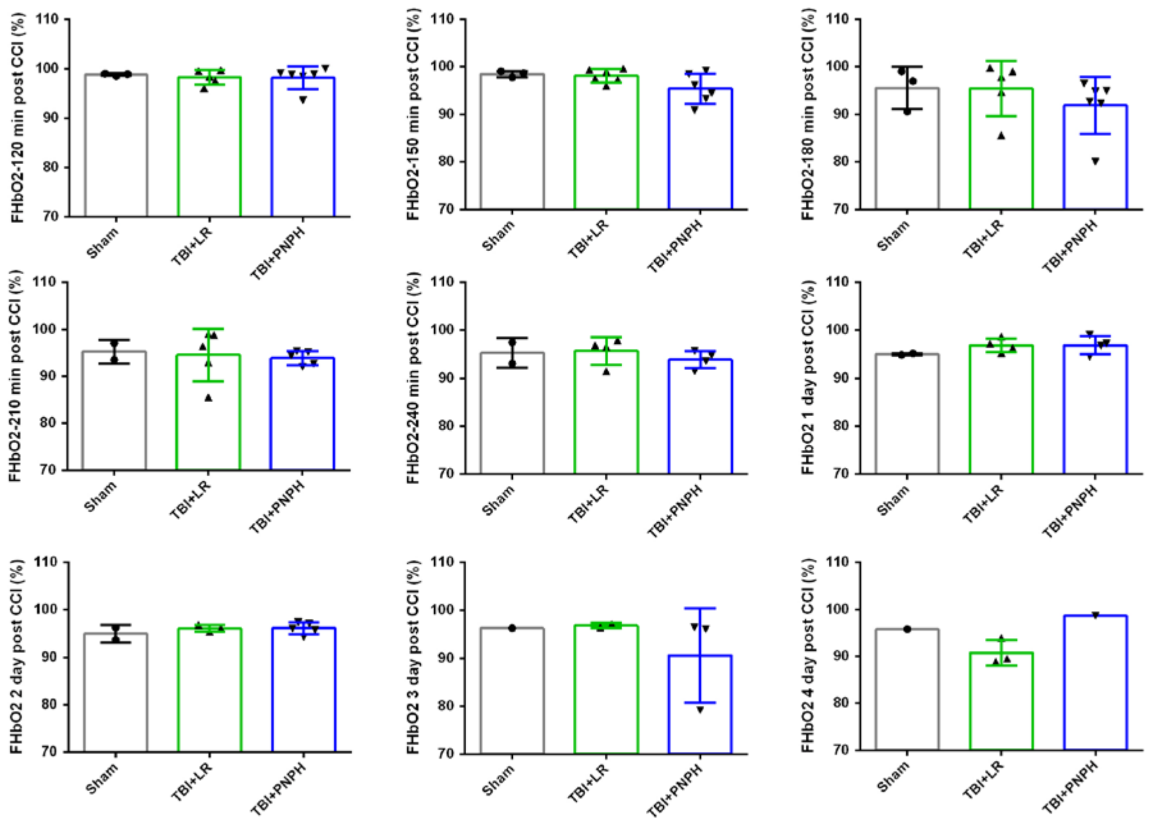

Supplemental Figure 8. Individual data and mean ±SD of the percent of arterial blood hemoglobin carrying oxygen (FHbO2) in the TBI alone experiment at 120, 150, 180, 210 and 240 minutes and at 1, 2, 3, and 4 days after TBI in groups resuscitated with 10 ml/kg of lactated Ringer's solution (LR) or 10 ml/kg of PNPH (infusion started at 120 minutes after TBI) and at equivalent times in a Sham surgery group. There were no significant differences among groups by one-way ANOVA at any time point; *n* = 3, 6, and 6 for Shams, LR, 10 ml/kg LR, and 10 ml/kg PNPH groups, respectively, at 120 min. Note that 2 arterial catheters stopped working by 120 min in the Sham group resulting in a small sample size, and that the sample sizes decreased after 120 min in all groups as pigs became ambulatory and too active to obtain valid readings or the subcutaneously-routed catheter became inoperable.

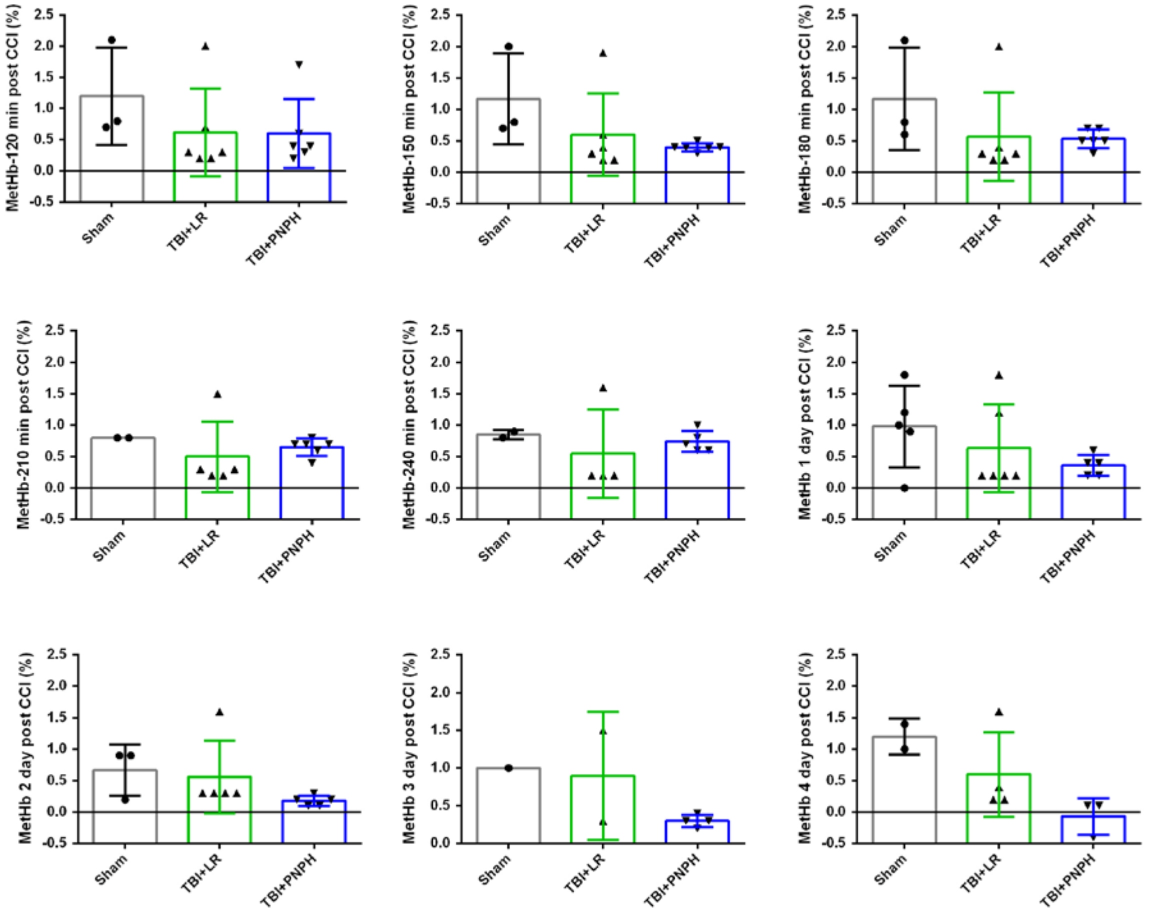

Supplemental Figure 9. Individual data and mean ±SD of the percent of blood methemoglobin (metHb) in the TBI alone experiment at 120, 150, 180, 210 and 240 minutes and at 1, 2, 3, and 4 days after TBI in groups resuscitated with 10 ml/kg of lactated Ringer's solution (LR) or 10 ml/kg of PNPH (infusion started at 120 minutes after TBI) and at equivalent times in a Sham surgery group. There were no significant differences among groups by one-way ANOVA at any time point; *n* = 3, 6, and 6 for Shams, LR, 10 ml/kg LR, and 10 ml/kg PNPH groups, respectively, at 120 min. Note that 2 arterial catheters stopped working by 120 min in the Sham group resulting in a small sample size, and that the sample sizes decreased after 120 min in all groups as pigs became ambulatory and too active to obtain valid readings or the subcutaneously-routed catheter became inoperable. In some cases, the venous Hb concentration was used at Days 1–4.

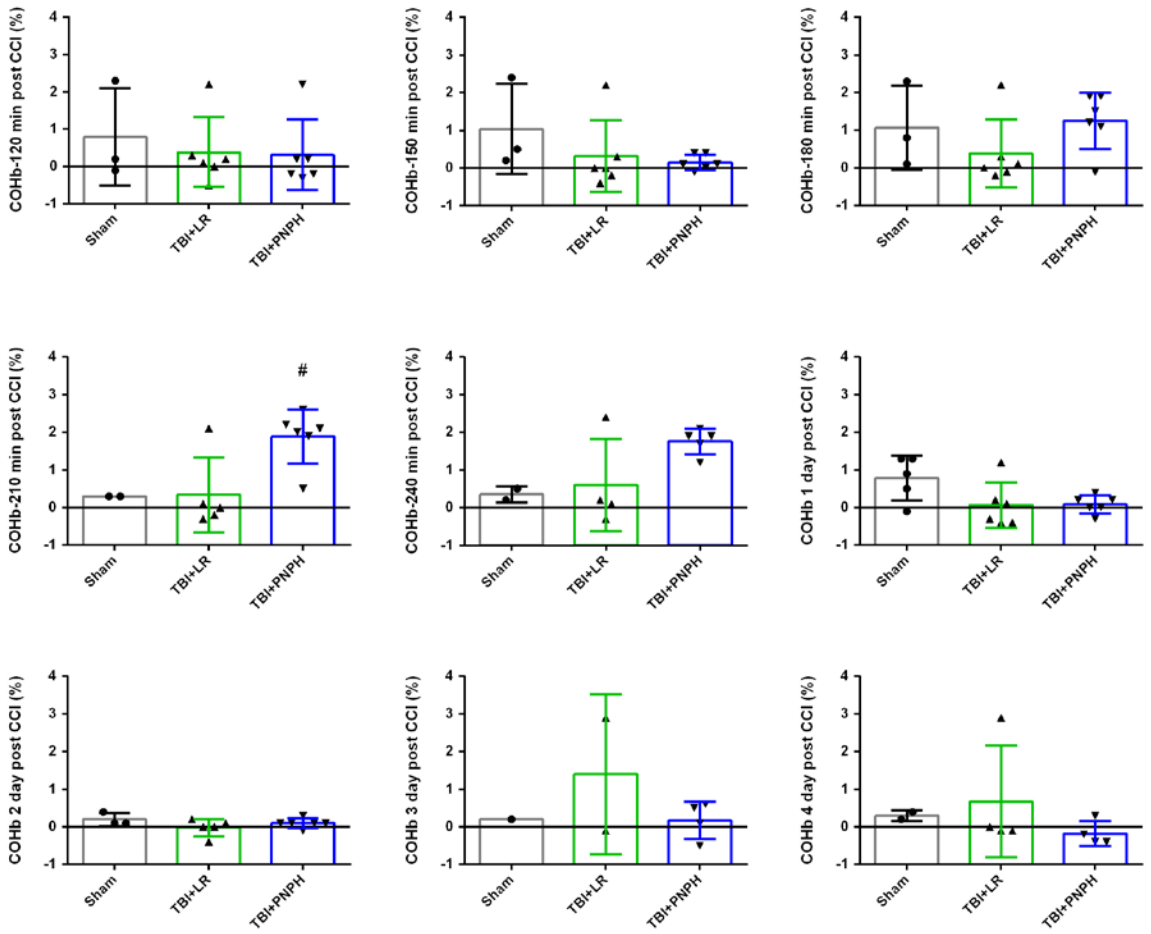

Supplemental Figure 10. Individual data and mean ±SD of the percent of whole blood carboxyhemoglobin (COHb)in the TBI alone experiment at 120, 150, 180, 210 and 240 minutes and at 1, 2, 3, and 4 days after TBI in groups resuscitated with 10 ml/kg of lactated Ringer's solution (LR) or 10 ml/kg of PNPH (infusion started at 120 minutes after TBI) and at equivalent times in a Sham surgery group. Data analyzed with one-way ANOVA and the Holm-Sidak procedure: # P <0.05 versus LR group; n = 3, 6, and 6 for Shams, LR, 10 ml/kg LR, and 10 ml/kg PNPH groups, respectively, at 120 min. Note that 2 arterial catheters stopped working by 120 min in the Sham group resulting in a small sample size, and that the sample sizes decreased after 120 min in all groups as pigs became ambulatory and too active to obtain valid readings or the subcutaneously-routed catheter became inoperable. In some cases, the venous Hb concentration was used at Days 1–4.
